# Abstracts from the 3rd International Severe Asthma Forum (ISAF)

**DOI:** 10.1186/s13601-017-0149-8

**Published:** 2017-05-24

**Authors:** M. E. Ketelaar, K. Van De Kant, F. N. Dijk, E. M. M. Klaassen, N. Grotenboer, M. C. Nawijn, E. Dompeling, G. H. Koppelman, Clare Murray, Philip Foden, Lesley Lowe, Hannah Durrington, Adnan Custovic, Angela Simpson, Andrew J. Simpson, Dominick E. Shaw, Ana R. Sousa, Louise J. Fleming, Graham Roberts, Ioannis Pandis, Aruna T. Bansal, Julie Corfield, Scott Wagers, Ratko Djukanovic, Kian Fan Chung, Peter J. Sterk, Jorgen Vestbo, Stephen J. Fowler, S. J. Tebbutt, A. Singh, C. P. Shannon, Y. W. Kim, C. X. Yang, G. M. Gauvreau, J. M. Fitzgerald, L. P. Boulet, P. M. O’Byrne, N. Begley, A. Loudon, D. W. Ray, Selene Baos, Lucía Cremades, David Calzada, Carlos Lahoz, Blanca Cárdaba, Kewal Asosingh, Chris Lauruschkat, Kimberly Queisser, Nicholas Wanner, Kelly Weiss, Weiling Xu, Serpil Erzurum, Milena Sokolowska, Li-Yuan Chen, Yueqin Liu, Asuncion Martinez-Anton, Carolea Logun, Sara Alsaaty, Rosemarie Cuento, Rongman Cai, Junfeng Sun, Oswald Quehenberger, Aaron Armando, Edward Dennis, Stewart Levine, James Shelhamer, Kilyong Choi, Snezhina Lazova, Penka Perenovska, Dimitrinka Miteva, Stamatios Priftis, Guergana Petrova, Vassil Yablanski, Evgeni Vlaev, Hristina Rafailova, Takashi Kumae, L. J. Holmes, J. Yorke, D. M. Ryan, Sasawan Chinratanapisit, Khlongtip Matchimmadamrong, Jitladda Deerojanawong, Wissaroot Karoonboonyanan, Paskorn Sritipsukho, Vania Youroukova, Denitsa Dimitrova, Yanina Slavova, Spaska Lesichkova, Iren Tzocheva, Snezhana Parina, Svetla Angelova, Neli Korsun, Mihai Craiu, Iustina Violeta Stan, Matea Deliu, Tolga Yavuz, Matthew Sperrin, Umit M. Sahiner, Danielle Belgrave, Cansin Sackesen Sackesen, Ömer Kalayci, Petar Velikov, Tsvetelina Velikova, Ekaterina Ivanova-Todorova, Kalina Tumangelova-Yuzeir, Dobroslav Kyurkchiev, Spyridon Megremis, Bede Constantinides, Alexandros Georgios Sotiropoulos, Paraskevi Xepapadaki, David Robertson, Nikolaos Papadopoulos, Maxim Wilkinson, Craig Portsmouth, David Ray, Royston Goodacre, Anna Valerieva, Irina Bobolea, Daiana Guillén Vera, Gabriel Gonzalez-Salazar, Carlos Melero Moreno, Consuelo Fernandez Rodriguez, Natividad De Las Cuevas Moreno, R. Wang, I. Satia, R. Niven, J. A. Smith, T. Southworth, J. Plumb, V. Gupta, J. Pearson, I. Ramis, M. D. Lehner, M. Miralpeix, D. Singh, Imran Satia, Mark Woodhead, Paul O’Byrne, Jaclyn Ann Smith, Cecilia Forss, Peter Cook, Sheila Brown, Freya Svedberg, Katherine Stephenson, Margherita Bertuzzi, Elaine Bignell, Malin Enerbäck, Danen Cunoosamy, Andrew Macdonald, Caini Liu, Liang Zhu, Kiochi Fukuda, Cunjin Zhang, Suidong Ouyang, Xing Chen, Luke Qin, Suguna Rachakonda, Mark Aronica, Jun Qin, Xiaoxia Li, Marie-Chantal Larose, Anne-Sophie Archambault, Véronique Provost, Jamila Chakir, Michel Laviolette, Nicolas Flamand, Nicola Logan, Dominik Ruckerl, Judith E. Allen, Tara E. Sutherland, E. Hamelmann, C. Vogelberg, S. Goldstein, G. E. Azzi, M. Engel, R. Sigmund, S. J. Szefler, Raquel Mesquita, Luis Coentrão, Rui Veiga, José-Artur Paiva, Roberto Roncon-Albuquerque, Wendy Vargas Porras, Ana González Moreno, Jesus Macías Iglesias, Gustavo Córdova Ramos, Yesenia Peña Acevedo, Miguel Angel Tejedor Alonso, Maria Del Mar Moro Moro, Irena Krcmova, Jakub Novosad, Nicola Alexander Hanania, Marc Massanari, Heike Hecker, Eric Kassel, Craig Laforce, Kathy Rickard, Sanne Snelder, Gert-Jan Braunstahl, T. L. Jones, D. Neville, E. R. Heiden, E. Lanning, T. Brown, H. Rupani, K. S. Babu, A. J. Chauhan, M. Y. Eldegeir, A. A. Chapman, M. Ferwana, M. Caldron

**Affiliations:** 1 Beatrix Children’s Hospital, Groningen Research Institute for Asthma and COPD (GRIAC), University Medical Center Groningen, Groningen, The Netherlands; 2 Department of Pediatric Pulmonology, School for Public Health and Primary Care (CAPHRI), Maastricht University Medical Center (MUMC+), Maastricht, The Netherlands; 3 Department of General Practice, School for Public Health and Primary Care (CAPHRI), Maastricht University Medical Center (MUMC+), Maastricht, The Netherlands; 4 Department of Pathology and Medical Biology, Laboratory of Experimental Pulmonology and Inflammation Research (EXPIRE), Groningen Research Institute for Asthma and COPD (GRIAC), University Medical Center Groningen, Groningen, The Netherlands; 5 University of Manchester, Manchester, UK; 6 Imperial College, London, UK; 7 Division of Infection, Immunity and Respiratory Medicine, School of Biological Sciences, The University of Manchester and University Hospital of South Manchester, NHS Foundation Trust, Manchester, UK; 8 Respiratory Research Unit, University of Nottingham, Nottingham, UK; 9 Respiratory Therapeutic Unit, GSK, Stockley Park, London, UK; 10 National Heart and Lung Institute, Imperial College, London, UK; 11 Biomedical Research Unit, Clinical and Experimental Sciences and Human Development and Health, NIHR Southampton Respiratory, Southampton, UK; 12 Data Science Institute, South Kensington Campus, Imperial College London, London, UK; 13 Acclarogen Ltd, St John’s Innovation Centre, Cambridge, UK; 14 AstraZeneca R&D, Mölndal, Sweden; 15 BioSci Consulting, Maasmechelen, Belgium; 16 Dept of Respiratory Medicine, Academic Medical Centre, University of Amsterdam, Amsterdam, The Netherlands; 17 University of British Columbia, Vancouver, Canada; 18 PROOF Centre of Excellence, Vancouver, Canada; 19 McMaster University, Hamilton, Canada; 20 Université Laval, Québec City, Canada; 21 IIS-Fundación Jiménez Díaz-UAM, Madrid, Spain; 22 Ciber de Enfermedades Respiratorias CIBERES, Madrid, Spain; 23 Cleveland Clinic, Cleveland, OH USA; 24 Critical Care Medicine Department, Clinical Center, NIH, Bethesda, MD USA; 25 Swiss Institute of Allergy and Asthma Research, University of Zurich, Zurich, Switzerland; 26 CK-CARE, Davos, Switzerland; 27 Laboratory of Asthma and Lung Inflammation, Cardiovascular and Pulmonary Branch, National Heart, Lung and Blood Institute, NIH, Bethesda, MD USA; 28 Department of Medicine, Department of Pharmacology, San Diego, CA USA; 29 Department of Chemistry and Biochemistry, San Diego, CA USA; 30 Seoul Medical Center, Seoul, South Korea; 31 Medical University, Pediatric Clinic, UMHAT Alexandrovska, Sofia, Bulgaria; 32 Medical University, Sofia, Bulgaria; 33 Orthopedics Department, Tokuda Hospital, Sofia, Bulgaria; 34 Research Institute, Tokyo University of Agriculture, Tokyo, Japan; 35 University Hospital of South Manchester & University of Manchester, Manchester, UK; 36 University Hospital of South Manchester, Manchester, UK; 37 Division of Allergy and Immunology, Department of Pediatrics, Bhumibol Adulyadej Hospital, Bangkok, Thailand; 38 Division of Respiratory Disease and Intensive Care, Department of Pediatrics, Faculty of Medicine, Chulalongkorn University, Bangkok, Thailand; 39 Department of Pediatrics, Bhumibol Adulyadej Hospital, Bangkok, Thailand; 40 Center of Excellence in Applied Epidemiology, Thammasat University, Bangkok, Thailand; 41 Clinical Center of Pulmonary Diseases; SHATPD “St. Sofia”, Medical Faculty of Medical University of Sofia, Sofia, Bulgaria; 42 Department of Clinical Immunology, Medical Faculty of Medical University of Sofia, University Hospital Alexandrovska, Sofia, Bulgaria; 43 Pediatric Clinic, University Hospital Alexandrovska, Medical University, Sofia, Bulgaria; 44 National Centre of Infectious and Parasitic Diseases, National Laboratory Influenza and ARD, Sofia, Bulgaria; 45 INSMC Alessandrescu-Rusescu, Bucharest, Romania; 46 Institute of Population Health, Health e-Research Centre, University of Manchester, Manchester, UK; 47 Division of Pediatric Allergy, GATA Military School of Medicine, Ankara, Turkey; 48 Centre for Health Informatics, Institute of Population Health, University of Manchester, Manchester, UK; 49 Division of Pediatric Allergy, Hacettepe University School of Medicine, Ankara, Turkey; 50 Faculty of Medicine, Department of Medicine, Imperial College London, London, UK; 51 Koç University Hospital, Istanbul, Turkey; 52 Department of Paediatrics, Imperial College London, London, UK; 53 Pediatric Allergy and Asthma Unit, Hacettepe University School of Medicine, Ankara, Turkey; 54 Laboratory of Clinical Immunology, University Hospital St. Ivan Rilski, Medical University, Sofia, Bulgaria; 55 University of Athens, Athens, Greece; 56 Division of Infection, Immunity and Respiratory Medicine, Faculty of Biology, Medicine and Health, University of Manchester, Manchester, UK; 57 Division of Metabolism and Gastroenterology, Faculty of Biology, Medicine and Health, University of Manchester, Manchester, UK; 58 School of Chemistry, Manchester Institute of Biotechnology, University of Manchester, Manchester, UK; 59 Manchester Academic Health Science Centre, and NIHR Respiratory and Allergy Clinical Research Facility, University Hospital of South Manchester, University of Manchester, Manchester, UK; 60 Department of Clinical Laboratory and Clinical Immunology, University Hospital, Medical Faculty of Medical University of Sofia, Sofia, Bulgaria; 61 Clinic of Allergy and Asthma, University Hospital Alexandrovska, Medical Faculty of Medical University of Sofia, Sofia, Bulgaria; 62 Hospital 12 de octubre, i+12 Research Institute, Madrid, Spain; 63 Almirall S. A., Barcelona, Spain; 64 Manchester Collaborative Centre for Inflammation Research, Manchester, UK; 65 Manchester Fungal Infection Group, Manchester, UK; 66 Saraburi Hospital, Saraburi, Thailand; 67 CRIUCPQ, Québec, Canada; 68 Institute of Immunology and Infection Research, University of Edinburgh, Edinburgh, UK; 69 Faculty of Biology, Medicine & Health, University of Manchester, Manchester, UK; 70 Manchester Collaborative Centre for Inflammation Research, University of Manchester, Manchester, UK; 71 Children’s Center, Evangelisches Krankenhaus Bielefeld, and Allergy Center, Ruhr University, Bochum, Germany; 72 University Hospital Carl Gustav Carus, Technical University of Dresden, Dresden, Germany; 73 Island Medical Research, Rockville Centre, New York City, NY USA; 74 Boehringer Ingelheim Pharma GmbH & Co. KG, Ingelheim Am Rhein, Germany; 75 Boehringer Ingelheim Pharma GmbH & Co. KG, Biberach An Der Riss, Germany; 76 Children’s Hospital of Colorado, University of Colorado School of Medicine, Aurora City, CO USA; 77 Internal Medicine Department, Centro Hospitalar de São João, Porto, Portugal; 78 Emergency and Intensive Care Medicine Department, Centro Hospitalar de São João, Porto, Portugal; 79 University Hospital Foundation Alcorcón, Madrid, Spain; 80 Institute of Clinical Immunology and Allergy, University Hospital, Hradec Kralove, Czechia; 81 Baylor College of Medicine, Houston, TX USA; 82 Circassia, Raleigh, NC USA; 83 North Carolina Clinical Research, Raleigh, NC USA; 84 Sint Franciscus Gasthuis, Rotterdam, The Netherlands; 85 Portsmouth Hospitals NHS Trust, Portsmouth, UK; 86 National Guard Hospital, Dammam, Saudi Arabia; 87 King Saud University, Riyadh, Saudi Arabia

## ORAL ABSTRACT SESSION 1—Asthma: from mechanisms to management

### O01 Serum IL-1RL1-A levels predict an eosinophilic subtype of asthma in preschool wheezing children

#### M. E. Ketelaar^1^, K. Van De Kant^2^, F. N. Dijk^1^, E. M. M. Klaassen^3^, N. Grotenboer^4^, M. C. Nawijn^4^, E. Dompeling^2^, G. H. Koppelman^1^

##### ^1^University Medical Center Groningen, Beatrix Children’s Hospital, Groningen Research Institute for Asthma and COPD (GRIAC), Groningen, The Netherlands; ^2^Department of Pediatric Pulmonology, School for Public Health and Primary Care (CAPHRI), Maastricht University Medical Center (MUMC+), Maastricht, The Netherlands; ^3^Department of General Practice, School for Public Health and Primary Care (CAPHRI), Maastricht University Medical Center (MUMC+), Maastricht, The Netherlands; ^4^University Medical Center Groningen, Department of Pathology and Medical Biology, Laboratory of Experimental Pulmonology and Inflammation Research (EXPIRE), Groningen Research Institute for Asthma and COPD (GRIAC), Groningen, The Netherlands

**Correspondence**: Maria Elizabeth Ketelaar - m.e.ketelaar@student.rug.nl

*Clinical and Translational Allergy* 2017, **7(Suppl 2)**:O01

**Introduction**: Respiratory symptoms are common in preschool children. However, which of these wheezers will develop asthma at school age, and what phenotype they will develop remains difficult to predict. Current models such as the asthma prediction index (API) are based on clinical parameters and have only modest predictive accuracy. Expression levels of well replicated asthma genes could potentially form novel biomarkers for asthma prediction. *IL1RL1* is an asthma susceptibility gene, and has also been linked to eosinophilia.

Therefore, we hypothesized that expression levels of *IL1RL1* in the form of soluble IL-1RL1-a measured in serum from wheezing preschool children contribute to the prediction of asthma at school age. Moreover, since *IL1RL1* was previously associated with blood eosinophilia, our second aim was to determine whether serum IL-1RL1-a levels predict eosinophilic asthma.

**Method**: We used logistic predictive modeling in a prospective Dutch birth cohort (n = 202 wheezers), and calculated the area under the curve (AUC) of the sensitivity/1-specificity curves of potential models.

**Results**: Neither IL-1RL1-a serum levels at age 2–3 years alone nor its combination with the API had predictive value for doctors’ diagnosed asthma at age 6y (*IL*-*1RL1*-*a alone:* AUC = 0.50 [95 CI 0.41–0.59, P = 0.98], *API* + *IL*-*1RL1*-*a:* AUC = 0.57 [95 CI 0.49–0.66, P = 0.12]).

However, IL-1RL1-a serum levels at age 2–3 years correlated with the severity of airway eosinophilia (determined by levels of exhaled fraction of NO, [FeNO]) in children who had developed asthma at age 6y (Pearson’s R = −0.24, P = 0.046, N = 59). Logistic predictive modeling of eosinophilic asthma at age 6y (asthma with FeNO ≥ 20 ppb) showed that IL-1RL1-a serum levels itself and in combination with the API could predict this eosinophilic subphenotype of asthma (*IL*-*1RL1*-*a alone:* AUC = 0.65 [95 CI 0.52–0.79, P = 0.04], *API* + *IL*-*1RL1*-*a:* AUC = 0.70 [95 CI 0.56–0.84, P = 0.01]). Interestingly, IL-1RL1-a levels had a negative direction of effect.

**Conclusion**: Our study shows that serum IL-1RL1–a levels measured in wheezing children at age 2–3 years do not predict doctors’ diagnosed asthma as general phenotype at age 6 years, but negatively predict an eosinophilic subphenotype of asthma.

This suggests that IL-1RL1 might play a protective role in the development of eosinophilia in children who experience asthma at school age and implies that IL-1RL1 targeted therapy could rather be further explored in the subphenotype of asthmatic children with predominant eosinophilic inflammation.

**Keywords**: Childhood Asthma, Eosinophilic Asthma, Prediction, IL-1RL1, Serum

**Figure:**
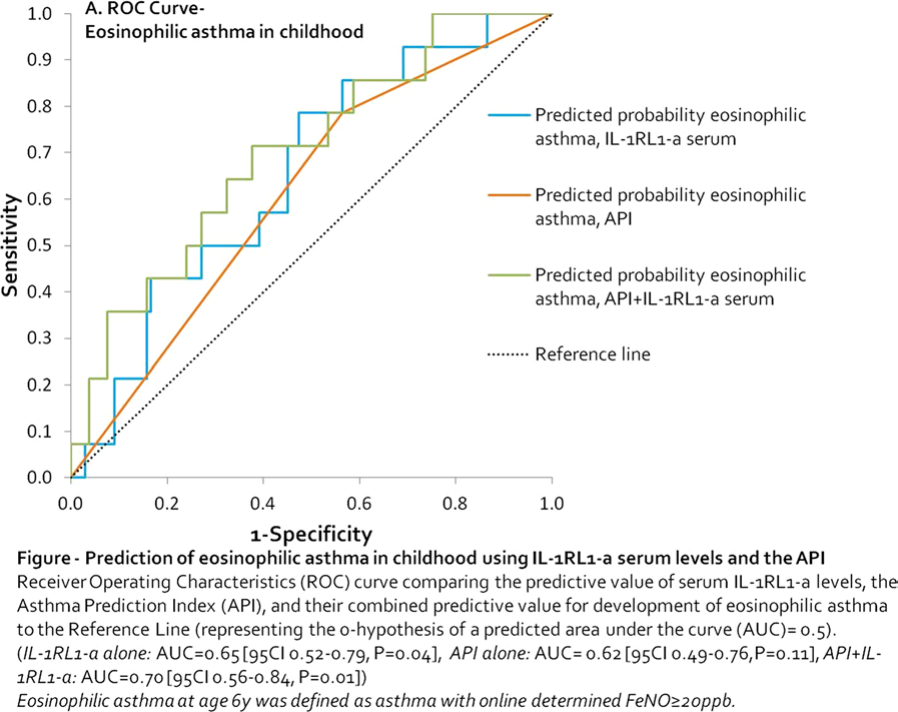
Figure 1 Prediction of eosinophilic childhood using IL-1 RL1-a serum levels and the API

### O03 Diagnosing asthma in symptomatic children using lung function: evidence from a birth cohort study

#### Clare Murray^1^, Philip Foden^1^, Lesley Lowe^1^, Hannah Durrington^1^, Adnan Custovic^2^, Angela Simpson^1^

##### ^1^University of Manchester, Manchester, United Kingdom; ^2^Imperial College, London, United Kingdom

**Correspondence**: Angela Simpson - angela.simpson@manchester.ac.uk

*Clinical and Translational Allergy* 2017, **7(Suppl 2)**:O03

**Introduction**: In the UK, new national draft guidance for the diagnosis of childhood asthma proposes algorithms based on four tests of lung function, each used as a dichotomous variable (FEV_1_/FVC ratio less than the lower limit of normal [LLN], bronchodilator reversibility [BDR] ≥12%, FeNO ≥ 35 ppb and PEFR variability). However, accuracy of these tests in diagnosing asthma in children is unknown, as the evidence is largely derived from studies of adults. Within the setting of a population-based birth cohort (Manchester Asthma and Allergy Study—MAAS), we investigated the value of FEV_1_/FVC, BDR and FeNO in diagnosing asthma in children.

**Method**: Using validated questionnaires we assessed study participants at age 16 years. Current asthma was defined as all three of: (1) doctor-diagnosed asthma ever, (2) wheezing in the previous 12 months and (3) current use of asthma treatment. We assigned children negative to all three features as non-asthmatic controls. Using ATS/ERS guidelines, we measured spirometry and FeNO (NIOX chemiluminescence analyser; Sweden). BDR was considered positive if FEV_1_ increased by ≥12% following administration of 400 mg of salbutamol. PEFR variability was not measured. To test the diagnostic algorithms simulating the clinic situation, we selected only children reporting recent symptoms of wheeze, cough or breathlessness who were not on regular inhaled corticosteroids (ICS).

**Results**: Of the 630 MAAS children with full data available, 163 reported recent symptoms, but were not using regular ICS; 34 of these met our definition of current asthma, with 55 as non-asthmatic controls. In the multivariable logistic regression analysis, increasing FeNO was associated with an increased risk of asthma (OR 1.02, 95% CI 1.01–1.04, p = 0.006), with a trend for FEV_1_/FVC ratio (OR 0.95, 95% CI 0.87–1.02, p = 0.17), and no association for BDR (p = 0.94). The proportion of those with each combination of positive tests is show as a Venn diagram (Figure 1). Of 58 children with three negative tests, 29.3% had current asthma, accounting for 50% of those with asthma. Only 5.9% of those with asthma were positive to all three tests.

**Conclusion**: Applying 3 tests of lung function to children with symptoms and a diagnosis of asthma failed to detect 50% of asthma cases. Proposed algorithms for the diagnosis of asthma in symptomatic children need to be tested prospectively.

**Keywords**: Asthma, Diagnosis, FeNO, Lung Function, Children

**Figure:**
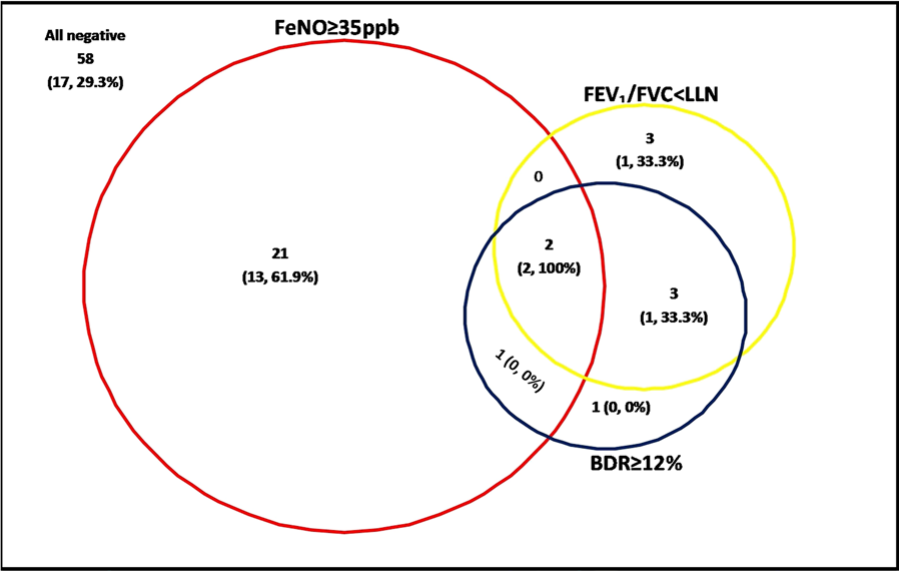
Figure 2 Venn diagram showing number of children with symptoms (n163) who were positive for each combination of tests

### O04 Treatable traits in the European U-BIOPRED adult severe asthma cohort

#### Andrew J. Simpson^1^, Dominick E. Shaw^2^, Ana R. Sousa^3^, Louise J. Fleming^4^, Graham Roberts^5^, Ioannis Pandis^6^, Aruna T. Bansal^7^, Julie Corfield^8^, Scott Wagers^9^, Ratko Djukanovic^5^, Kian Fan Chung^4^, Peter J. Sterk^10^, Jorgen Vestbo^1^, Stephen J. Fowler^1^

##### ^1^Division of Infection, Immunity and Respiratory Medicine, School of Biological Sciences, The University of Manchester and University Hospital of South Manchester, NHS Foundation Trust, Manchester, United Kingdom; ^2^Respiratory Research Unit, University of Nottingham, Nottingham, United Kingdom; ^3^Respiratory Therapeutic Unit, GSK, Stockley Park, London, United Kingdom; ^4^National Heart and Lung Institute, Imperial College, London, United Kingdom; ^5^NIHR Southampton RespiratoryBiomedical Research Unit, Clinical and Experimental Sciences and Human Development and Health, Southampton, United Kingdom; ^6^Data Science Institute, South Kensington Campus, Imperial College London, London, United Kingdom; ^7^Acclarogen Ltd, St John’s Innovation Centre, Cambridge, United Kingdom; ^8^AstraZeneca R&D, Mölndal, Sweden; ^9^BioSci Consulting, Maasmechelen, Belgium; ^10^Dept of Respiratory Medicine, Academic Medical Centre, University of Amsterdam, Amsterdam, The Netherlands

**Correspondence**: Andrew J. Simpson - Andrew.Simpson-2@Manchester.ac.uk

*Clinical and Translational Allergy* 2017, **7(Suppl 2)**:O04

**Introduction**: Individuals with severe asthma may remain uncontrolled and exacerbation-prone despite intensive guideline-directed treatment, and management options are limited. The concept of treatable traits, based on the identification of treatable disease-associated characteristics, may thus be a particularly useful framework in this context. In the Unbiased Biomarkers for the Prediction of Respiratory Disease Outcomes (U-BIOPRED) project we have recruited individuals with severe asthma (SA) under specialist care, and controls with mild to moderate asthma (MMA).

**Aim**: To identify and quantify treatable traits within the U-BIOPRED adult asthma cohorts.

**Method**: We defined criteria for treatable traits based on Agusti (*Eur Respir J,* 2016) and identified prevalence rates within the U-BIOPRED database. Chi Square tests were used to examine differences in frequency between individuals with SA and MMA

**Results**: Data from 509 individuals with asthma were included in the analysis; 421 with SA and 88 with MMA. Twenty-nine treatable traits were identified, including 13 pulmonary, 13 extra-pulmonary and three behavioral traits. Pulmonary treatable traits such as airflow limitation (SA 50% vs. MMA 6%, P < 0.001), reversibility (SA 58% vs. MMA 39%, P = 0.002), eosinophilia (SA 54% vs. MMA 43%, P = 0.067), exercise-induced asthma (SA 77% vs. MMA 54%, P = 0.002), allergic rhinitis (SA 47% vs. MMA 44%, P = 0.641), cough (SA 63% vs. MMA 19%, P < 0.001) and bronchitis (SA 51% Vs. MMA 16%, P = 0.000) were highly prevalent in the asthma cohorts, and typically more common in SA *versus* MMA. The most common extra-pulmonary treatable traits were; atopy (SA 74% vs. MMA 92%, P < 0.001), obesity (SA 30% vs. MMA 38%, P = 0.178), reflux (SA 36% vs. MMA 11%, P < 0.001), hypertension (SA 25% vs. MMA 9%, P = 0.001) and obstructive sleep apnea (SA 26% vs. MMA 11%, P = 0.003). Behavioral traits included low medication adherence (SA 39% vs. MMA 52%, P = 0.031) and smoking (SA 8%, smokers not eligible in MMA cohort). Participants with SA had mean (SD) 8 ± (2), and MMA 5 ± (2) treatable traits.

**Conclusion**: We have applied a new approach for the characterisation of severe asthma, based on treatable traits. In general these traits were found more commonly in severe than non-severe asthma; the very high prevalence of many of these traits suggests that there are potential targets for treatment even in such severe patients, and supports the need for specialist management.

**Keywords**: Phenotypes, Treatable Traits

## ORAL ABSTRACT SESSION 2—Molecular mechanisms

### O05 Discovery, development and validation of blood-based RNA biomarker panels to predict the late-phase asthmatic response

#### S. J. Tebbutt^1^, A. Singh^1^, C. P. Shannon^2^, Y. W. Kim^1^, C. X. Yang^1^, G. M. Gauvreau^3^, J. M. Fitzgerald^1^, L. P. Boulet^4^, P. M. O’Byrne^3^

##### ^1^University of British Columbia, Vancouver, Canada; ^2^PROOF Centre of Excellence, Vancouver, Canada; ^3^McMaster University, Hamilton, Canada; ^4^Université Laval, Québec City, Canada

**Correspondence**: Scott J. Tebbutt - scott.tebbutt@hli.ubc.ca

*Clinical and Translational Allergy* 2017, **7(Suppl 2)**:O05

**Introduction**: We have previously demonstrated significant molecular changes in the blood between mild allergic asthmatic individuals who develop isolated early responses (early responders, ERs) compared to those who also develop late-phase responses (dual responders, DRs) after allergen inhalation challenge. Identifying individuals likely to develop dual responses may aid in the screening of subjects for clinical trials that test drugs for the attenuation of the late-phase asthmatic response, which shares hallmark features of chronic disease. The objective of this study was to develop blood-based biomarker panels that could identify asthmatic individuals with high probability of developing a late-phase response.

**Method**: The discovery cohort consisted of 36 mild asthmatic subjects (15 ERs and 21 DRs) and a validation cohort consisted of 45 mild asthmatic subjects (9 ERs and 36 DRs). Blood samples were collected prior to allergen challenge. Following RNA extraction, total RNA (globin-/ribo-depleted) was sequenced using an Illumina HiSeq 2000 as 100 base paired-end reads. Both genome-guided datasets (UCSC genes, UCSC gene-isoforms, and Ensembl) and *de novo* assembled transcripts using the Trinity software were constructed. Top-ranked biomarker candidates were transferred to the high-precision and clinically-approved NanoString nCounter platform. Final biomarker panels were identified, and statistical algorithms were locked down prior to testing in the validated cohort.

**Results**: Predictive biomarker panels had classification performance (based on the area under the receiver operating curve, AUC) ranging between 60 and 70% in the discovery cohort. 87 transcripts identified on the RNA-Seq platform were transferred to NanoString Elements assay chemistry. The transcripts were split with respect to their dataset of origin and tested in the validation cohort. The UCSC gene-isoforms and Trinity biomarker panels had AUCs of 68% and 72%, respectively. The biomarker transcripts were enriched for biological pathways such as NF-ĸB signaling (up-regulated in DRs) and apoptosis, decoy receptors and formyl peptide receptors (down-regulated in DRs).

**Conclusion**: RNA transcript biomarker panels in the blood were successful at predicting asthmatic subjects likely to develop dual responses upon allergen inhalation challenge. Pathways and networks represented by these biomarker panels may reveal new avenues for therapeutic targeting of the pathobiology involved in chronic asthma.

**Keywords**: Predictive Biomarkers, RNA-Seq, NanoString Elements, Statistical Algorithms

### O06 The clock Gene REV-ERBalpha regulates airway hyperresponsiveness: implications for asthma

#### H. J. Durrington, N. Begley, A. Loudon, D. W. Ray

##### University of Manchester, Manchester, United Kingdom

**Correspondence**: Hannah J. Durrington - hannah.durrington@manchester.ac.uk

*Clinical and Translational Allergy* 2017, **7(Suppl 2)**:O06

**Introduction**: Asthma is an inflammatory disease with a strong circadian signature. Symptoms of asthma are worse overnight and in the early hours of the morning. Measurements of peak expiratory flow rate and forced expiratory volume in 1 s, are lower in early morning compared to afternoon. It is likely that circadian biology plays a role in the pathogenesis of asthma. A better understanding of the circadian nature of asthma may lead to better treatments through chronotherapy (taking medication at the most beneficial time of day). Clock genes control circadian rhythms within every cell in the body. The clock gene, REV-ERB alpha (NR1D1), connects the ‘core’ clock and the immune system. We employed a REV-ERB alpha global knockout mouse (*rev*-*erb alpha* ^−/−^, these mice retain circadian rhythmicity, but show loss of temporal variation of innate immunity) to investigate the effect of REV-ERB alpha on airway hyper-responsiveness (AHR). AHR is a key pathophysiological feature of asthma.

**Method**: All experimental procedures were carried out in accordance with the Animals (Scientific Procedures) Act,1986. *Rev*-*erb alpha* ^−/−^ mice were provided by Ueli Schibler (University of Geneva).

Measurements of dynamic resistance were performed using a Flexivent system (Scireq, Montreal, Canada). After induction of anaesthesia with an intrperitoneal injection of ‘Hypnorm’ (0.315 mg fentanyl; 10 mg fluanisone) and midazolam (5 mg) in water, at a dose of 0.1 ml/10 g, mice were tracheostomized and connected to the flexivent ventilator via an 18-guage needle. Mice were ventilated. Changes in resistance were measured in response to increasing concentrations of nebulized methacholine from 0 to 300 mg/ml. All measurements were made in the morning.

**Results**: *Rev*-*erb alpha* ^−/−^ mice exhibit significantly (p < 0.01) greater AHR to increasing concentrations of methacholine compared to their wildtype and heterozygote litter-mate controls (Figure 1).

**Conclusion**: *Rev*-*erb alpha* ^−/−^ mice retain circadian rhythmicity yet demonstrate increased AHR to methacholine (as is seen in human asthma). REV-ERB alpha may play an important role in the pathogenesis of asthma.

Next steps:

1. Investigate a time of day difference in AHR in *Rev*-*erb alpha* ^−/−^ mice
2. Expose Rev-erb alpha^−/−^ mice to the house dust mite model of asthma
3. Employ REV-ERB alpha ligands to ‘modify’ the phenotype

**Keywords**: Airway Hyperresponsiveness, Rev-Erb Alpha, NR1D1, Clock Gene

**Figure:**
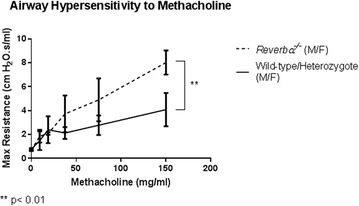
Figure 3 Airway Hypersensitivity to Methacholine

### O07 Asthma biomarkers: methylation and correlation with functional parameters

#### Selene Baos^1^, Lucía Cremades^1^, David Calzada^1^, Carlos Lahoz^2^, Blanca Cárdaba^2^

##### ^1^IIS-Fundación Jiménez Díaz-UAM, Madrid, Spain; ^2^IIS-Fundación Jiménez Díaz-UAM; Ciber de Enfermedades Respiratorias, CIBERES, Madrid, Spain

**Correspondence**: Selene Baos - selene.baos@fjd.es

*Clinical and Translational Allergy* 2017, **7(Suppl 2)**:O07

**Introduction**: In a previous study (submitted to publish) we defined specific genes related with asthma and allergic diseases, by studying the gene-expression of 94 genes in a population composed by 4 groups of subjects: healthy control, nonallergic asthmatic, asthmatic allergic and nonasthmatic allergic patients. The analysis of differential gene-expression between control and patients with nonallergic asthma revealed a set of statistically relevant genes mainly associated with the disease’s severity: IL10, MSR1, PHLDA1, SERPINB2, CHI3L1, IL8, and PI3. In this project we analyzed if methylation takes part in the regulation of the gene-expression of these potential asthma biomarkers and their correlation with protein expression and functional parameters (%FVE1,  %FVC and  %PBD).

**Method**: DNA extracted from PBMCs of control and nonallergic asthma subjects was treated with sodium bisulfite and amplified by PCR with primers designed to amplify CpG islands near the promotor region of the most significant genes: MSR1 (5 CpG islands), SERPINB2 (5), PHLDA1 (35), CHI3L1 (8) and PI3 (5). The methylation analysis was done with the Sequenom EpiTYPER approach. Protein quantification was determined by ELISA or Western Blot. Statistical analyses were performed with Graph-Pad program.

**Results**: The methylation analysis showed statistically significant differences between groups in 4 genes: MSR1, PHLDA1, CHI3L1 and PI3. The results of the methylation correlated with the gene-expression, the protein levels and respiratory parameters, specifically in MSR1, CHI3L1 and PI3.

**Conclusion**: A possible regulatory mechanism of molecular biomarkers of asthma has been defined, being methylation a possible key factor for the differential gene-expression of asthma patients.

**Keywords**: DNA Methylation, Biomarkers, Epigenetics

### O08 Critical role for Arginine metabolism in a combined Th2 and Th17 airway inflammation in the house dust mite model

#### Kewal Asosingh, Chris Lauruschkat, Kimberly Queisser, Nicholas Wanner, Kelly Weiss, Weiling Xu, Serpil Erzurum

##### Cleveland Clinic, Cleveland, Ohio, United States

**Correspondence**: Kewal Asosingh - asosink@ccf.org

*Clinical and Translational Allergy* 2017, **7(Suppl 2)**:O08

**Introduction**: Inducible nitric oxide synthase (iNOS) and arginase-2 (ARG2) share a common substrate arginine. We recently showed that both enzymes are highly expressed in asthmatic airway epithelial cells where ARG2 plays a critical role in the regulation of mitochondrial bioenergetic driven Th2 response, as shown by the development of severe eosinophilic airway inflammation in mice deficient for *ARG*-*2* (*ARG*-*2* KO). The role of iNOS and ARG2 in a combined Th2 and Th17 response is unknown. Here we hypothesize that Th17 airway inflammation is also regulated by ARG2 driven metabolism.

**Method**: Commercially available Wildtype (WT) and *iNOS* knockout (KO) were used. *ARG2* KO was donated to us and *ARG2iNOS* double KO (dKO) strain was generated in house. Standard house dust mite extract (HDME) mouse model of Th2/Th17 asthma endotype was used. Airway inflammation was quantified by analysis of inflammatory cells in bronchoalveolar lavage fluid (BALF). Airway angiogenic remodeling was measured by quantification of lung microvessel density on tissue sections stained for the endothelial marker von Willebrand Factor. Epithelial and immune cell cytokines were measured by ELISA or flow cytometric multiplex assay.

**Results**: HDME induced airway inflammation in all genotypes, but was the highest in *ARG2* KO as showed by increased number inflammatory cells in the BALF. Both eosinophilic (Th2) and neutrophilic (Th17) inflammation peaked in *ARG2* KO mice but were attenuated in *ARG2iNOS* dKO animals. Airway inflammation was similar among HDME exposed WT, *iNOS* KO and *ARG2iNOS* dKO mice. Angiogenic airway remodeling was also highest in *ARG2* KO mice and comparable among WT, *iNOS* KO and *ARG2iNOS* dKO genotypes. Airway epithelial or immune cell-derived Th2 cytokines IL-5 and eotaxin-2 and, Th17 cytokine IL-17 were maximal in lungs of HDME exposed *ARG2* KO mice, but decreased in *ARG2iNOS* dKO.

**Conclusion**: The data show that *ARG2* deficiency induces a severe combined Th2 and Th17 airway inflammation. Deletion of *iNOS* in addition to *ARG2* inhibited disease severity, but *iNOS* deletion alone, had no effect, indicating that ARG2 is the most critical of the two arginine consuming enzymes in the regulation of inflammation and angiogenesis in asthma. Overall, the results expand our previous findings by demonstrating that in addition to Th2, Th17 endotype of airway inflammation is also critically regulated by arginine metabolism

**Keywords**: Arginine, Metabolism, Th2, Th17, Mitochondria

### O09 Hyaluronan-induced lipid mediators influence antiviral and antibacterial immunity in severe asthmatics

#### Milena Sokolowska^1^, Li-Yuan Chen^2^, Yueqin Liu^2^, Asuncion Martinez-Anton^2^, Carolea Logun^2^, Sara Alsaaty^2^, Rosemarie Cuento^3^, Rongman Cai^2^, Junfeng Sun^2^, Oswald Quehenberger^4^, Aaron Armando^5^, Edward Dennis^5^, Stewart Levine^3^, James Shelhamer^2^

##### ^1^Critical Care Medicine Department, Clinical Center, NIH; Swiss Institute of Allergy and Asthma Research, University of Zurich; CK-CARE, Davos, Switzerland; ^2^Critical Care Medicine Department, Clinical Center, NIH, Bethesda, Maryland, United States; ^3^Laboratory of Asthma and Lung Inflammation, Cardiovascular and Pulmonary Branch, National Heart, Lung and Blood Institute, NIH, Bethesda, Maryland, United States; ^4^Department of Medicine, Department of Pharmacology, San Diego, California, United States; ^5^Department of Chemistry and Biochemistry, San Diego, California, United States

**Correspondence**: Milena Sokolowska - milena.sokolowska@siaf.uzh.ch

*Clinical and Translational Allergy* 2017, **7(Suppl 2)**:O09

**Introduction**: Hyaluronan (HA) is the major glycosaminoglycan in the extracellular matrix involved in the pathogenesis of asthma and other chronic inflammatory diseases. During inflammation in asthma there is an increased breakdown of HA, resulting in the local and systemic accumulation of low molecular weight (LMW) HA. Eicosanoids, derived from cytosolic phospholipase A_2_ group IVA (cPLA_2_alpha) activation, are potent lipid mediators also attributed to acute and chronic inflammation.

**Method**: We investigated the effect of LMW HA on the lipidomic profile and global gene expression and their functional interactions in peripheral blood mononuclear cells of patients with mild-to-moderate (n = 7) and severe asthma (n = 6) as compared to controls (n = 6).

**Results**: We found that LMW HA increased production of 68 unique lipid species, among which PGE_2_, PGB_2_, PGD_2_, 15-HETE, TxB_2_, 11(12)-EET, 14(15)-EET, 13-HOTrE(y) and 16(17)-EpDPE were significantly upregulated only in severe asthmatics. We also performed a genome-wide expression analysis of LMW HA signaling, confirming its highly immunostimulatory potential. However, in severe asthmatics the LMW HA-induced global gene expression profile showed a comprehensive impairment in interferon signaling, cell apoptosis and cell movement, leading to diminished antiviral and antibacterial responses. We confirmed these findings at the protein level, finding that LMW HA-induced production of IL-12 p40, CXCL10, CXCL11 and CCL8 in severe asthmatics was markedly reduced. Importantly, upon cPLA_2_alpha inhibition, there was a significant decrease in lipid mediator production accompanied by a significant increase in IL-12 p40 and CXCL9 protein expressions in each phenotype.

**Conclusion**: In summary, we demonstrated here that fragmented hyaluronan increased production of several unique lipid species in severe asthmatics. Moreover, through analysis of the whole genome profile, captured simultaneously with the lipidomic profile, we observed decreases in antiviral gene and protein expression upon LMW HA treatment in severe asthmatics, which was partially reversed upon inhibition of lipid production. Therefore, our current findings provide new evidence on the connection between extracellular matrix, global lipid mediator production and decreased antiviral responses in severe asthma.

**Keywords**: Severe Asthma, Eicosanoid, Virus, Lipidomics, Transcriptomics

## POSTER DISCUSSION SESSION 1—Epidemiology

### P01 Fungal communities in house dust associated with the development of Atopic Dermatitis in infant

#### Kilyong Choi

##### Seoul Medical Center, Seoul, South Korea

**Correspondence**: Kilyong Choi - bestchoi9494@gmail.com

*Clinical and Translational Allergy* 2017, **7(Suppl 2)**:P01

**Introduction**: In our epidemiologic study, the effect of mold exposure was associated with the exacerbation or the development of atopic dermatitis (AD). Maternal exposure to mold during the pregnant periods or exposure to mold in early life particularly have an impact on the increased risk of AD development. However, its specific mechanism still remains unclear. In addition, dust is a repository and concentrator of many chemical and biological agents including fungi. The aims of this study were to identify the fungal microbiota (mycobiota) in house dust, and to investigate whether the mycobiota is associated with the development of AD.

**Method**: To identify the mycobiota in house dust, DNA pyrosequencing was performed targeting the internal transcribed spacer (ITS) region. Here, we investigated the composition of fungal communities in the dust samples from houses of 20 healthy and 20 AD infants diagnosed at 6 and/or 12 months.

**Results**: A total of 7 phyla and 399 fungal genera were distinguished in house dust. Fungal alpha diversity was similar between two groups. However, our results show that the different fungal communities between healthy and AD groups. The genera *Alternaria, Aspergillus, Fusarium* and *Candida* belonging to Ascomycota and known as the common allergen were higher abundant in dust samples for infants with AD. On the other hand, *Malassezia, Pleuotus* and *Trichosporon* belonging to Basidiomycota were less abundantin dust samples for infants with AD.

**Conclusion**: House dust from infants with AD show higher abundance of specific fungi known as allergens, which suggests that different fungal exposure from the dust may link to the development of AD in infancy.

**Keywords**: House Dust, Atopic Dermatitis, Infant, Fungal Communities

**Table:** Table 1 Library coverage estimations and sequence diversity of fungal ITS genes pyrosequencing

ID	Valid reads	OTUs	Chao1	Shannon	Simpson	Goods Lib. Coverage
S13-033-C	9475	474	769.5882	3.619653	0.114784	0.978786
S12-073-C	8588	456	567.125	4.541252	0.02198	0.985212
S12-067-C	10169	611	731.3051	4.629529	0.024318	0.983381
A13-033-C	9719	377	455.9153	3.690041	0.090025	0.99002
A12-080-C-22	7111	315	404.7581	3.541059	0.09324	0.985094
A12-072-C	7899	439	757.3333	3.991715	0.051364	0.97582
A12-054-C	9467	343	574.6667	3.859098	0.05143	0.985212
Y10-533-C	10273	520	775.6081	4.314013	0.038003	0.981018
C12-044-C	8788	382	518.1224	4.288241	0.030933	0.9868
Y12-016-C	11603	576	952.9615	4.045346	0.052539	0.979057
A13-047-C	9386	517	740.9474	4.419917	0.037477	0.98029
C13-065-C	11197	469	599.013	4.253351	0.033662	0.987318
A10-581-C	11167	530	693.4	4.24386	0.047244	0.984597
A10-609-C	12620	725	1126.857	4.347734	0.035431	0.976941
A12-037-C	11089	828	1181.979	4.64697	0.035575	0.970962
A13-069-C	12210	764	1144.971	4.907156	0.02687	0.976904
C13-067-C	10215	570	733.8298	4.774492	0.021033	0.98277
S12-072-C	6413	317	375.2632	3.910256	0.058516	0.987213
Y12-044-C	12008	638	949.3542	4.230534	0.070634	0.979597
A12-071-C	4753	544	746.4118	5.021128	0.018621	0.960867
Y12-051-C	7282	329	416.3061	4.209385	0.030832	0.987229
A10-575-C	9011	608	787.1	4.68377	0.025285	0.977916
A12-074-C	10195	410	549.2642	3.913959	0.060137	0.988033
A13-023-C	10885	366	425.7143	4.232292	0.035282	0.992926
A13-016-C	11250	573	778.8875	4.588823	0.026887	0.983822
C12-042-C	8323	341	639.5294	3.248262	0.102067	0.978974
A13-048-C-21	10255	504	647.3544	4.15016	0.049551	0.985275
S12-056-C	11210	940	1505.511	4.965526	0.03022	0.964585
S12-052-C	10900	578	787	3.604678	0.118101	0.980734
A12-051-C	11160	688	1004.469	4.548595	0.0311	0.977867
A12-036-C	11206	492	711.6133	3.816188	0.086481	0.983759
C13-033-C	11982	562	863.8889	4.367747	0.030685	0.982557
Y13-018-C	11885	496	632.2805	4.114662	0.045829	0.987379
A10-596-C	8604	438	726.0159	3.686982	0.075458	0.977801
S13-025-C	9653	503	786.5156	4.473125	0.025739	0.980213
S13-029-C	10485	580	815.6915	4.305426	0.03932	0.979876
C14-006-C	11607	633	941.9326	4.389317	0.04185	0.979754
C14-009-C	10267	528	823.5882	4.297914	0.050246	0.980423
S12-090-C	10219	480	665.2442	4.017626	0.053966	0.982484
S13-045-C	10677	421	560.2344	3.541324	0.118609	0.98745
Total	401206	20865

**Table:** Table 2 List of fungal genera and their relative abundance (%) in house dust samples from two groups

Taxon		Status		
Phylum	Genus	Health (n = 20)	AD (n = 20)	*p* Value
Ascomycota	*Alternaria*	7.9104	12.8797	0.079
Ascomycota	*Aspergillus*	4.1237	5.1118	0.705
Ascomycota	*Aureobasidium*	5.8441	5.0303	0.85
Ascomycota	*Candida*	0.7504	1.0536	0.725
Ascomycota	*Cladosporium*	7.8394	7.6885	0.725
Ascomycota	*Cyphellophora*	0.5648	0.6081	0.022
Ascomycota	*Exophiala*	0.3533	0.8299	0.957
Ascomycota	*Fusarium*	0.6404	1.3015	0.685
Ascomycota	*Leptosphaerulina*	1.4300	0.8881	0.291
Ascomycota	*Meyerozyma*	0.0367	1.7910	0.989
Ascomycota	*Nigrospora*	0.7219	0.5520	0.344
Ascomycota	*Penicillium*	2.7624	1.3681	0.978
Ascomycota	*Peyronellaea*	1.6187	2.7516	0.818
Ascomycota	*Phoma*	7.1393	6.3727	1
Ascomycota	*Pleospora*	0.4702	0.5722	0.487
Ascomycota	*Saccharomyces*	2.9811	1.0517	0.176
Basidiomycota	*Cryptococcus*	1.8277	2.1603	0.725
Basidiomycota	*Malassezia*	10.8815	4.4114	0.005
Basidiomycota	*Malasseziaceae_uc*	0.8520	1.2991	0.626
Basidiomycota	*Pleurotus*	3.2915	0.0260	0.005
Basidiomycota	*Rhodotorula*	1.5704	0.7741	0.725
Basidiomycota	*Trichosporon*	4.1956	0.3016	0.003

### P02 Cluster analysis in Bulgarian children with asthma

#### Snezhina Lazova^1^, Penka Perenovska^1^, Dimitrinka Miteva^1^, Stamatios Priftis^2^, Guergana Petrova^1^

##### ^1^Medical University, Pediatric clinic, UMHAT Alexandrovska, Sofia, Bulgaria; ^2^Medical University, Sofia, Bulgaria

**Correspondence**: Snezhina Lazova - snejina@lazova.com

*Clinical and Translational Allergy* 2017, **7(Suppl 2)**:P02

**Introduction**: Asthma is a complex heterogeneous disease that likely comprises several distinct disease phenotypes. Usually atopy presence or any allergic diseases could interfere and obstacle the control of asthma. Clustering approach has been used to classify heterogeneous asthma population into distinct phenotypes.

**Method**: For a period of 1 year we evaluated medical history data of 110 children with asthma aged 3 to 17 years. For all children we performed pulmonary function tests, nasal smears for eosinophil counts, drew blood for IgE against inhalation and food allergies antibodies detection and ACQ. IgE were detected with the predesigned kit EurolinePediatric and total IgE with Immunocap. Differences in clinical indices including ΔFEV1(pre- and post-bronchodilator) and nasal and blood biomarkers at enrollment among clusters were analyzed.

**Results**: Five distinct phenotypes were determined. Cluster 1 (n = 13): (non-atopic) the lowest IgE level, very low ACQ, median age of diagnosis and IgE levels. Cluster 2 (n = 45): (mixed) the highest BMI with the latest age of diagnosis and high ACQ and BDR levels. Cluster 3 (n = 33) (atopic) early diagnosis, highest BDR, highest ACQ score, highest total and specific IgE levels among the clusters. Cluster 4 (n = 12): (atopic) the highest Immunocap result, relatedly high BMI and IgE with median ACQ score among clusters. Cluster 5 (n = 7): (non-atopic) the earliest age for diagnosis, with the lowest BMI, the lowest ACQ score, and Immunocap result, with high BDR and median level of IgE among clusters.

The patients with severe asthma were over 65% of the patients in cluster 3. Only one severe asthma patient was in cluster 2, and none in the other three clusters.

**Conclusion**: We identified asthma phenotypes in Bulgarian children according IgE levels, ACQ score, BDR and age of diagnosis. Almost all of the severe asthma patients were in cluster 3 – with early diagnosis and highest IgE but not highest Immunocap results.

**Keywords**: Atopy, IgE, ACQ, Severe Asthma

### P03 Lung function parameters in asthma, severe asthma and scoliosis in children

#### Snezhina Lazova^1^, Vassil Yablanski^2^, Penka Perenovska^1^, Evgeni Vlaev^2^, Hristina Rafailova^1^, Stamatios Priftis^3^, Guergana Petrova^1^

##### ^1^Medical University, Pediatric cilinic, UMHAT Alexandrovska, Sofia, Bulgaria; ^2^Orthopedics department, Tokuda Hospital, Sofia, Bulgaria; ^3^Medical University, Sofia, Bulgaria

**Correspondence**: Snezhina Lazova - snejina@lazova.com

*Clinical and Translational Allergy* 2017, **7(Suppl 2)**:P03

**Introduction**: Pulmonary function testing (PFT) is of great importance in the evaluation of lung function. Spirometry is simple, noninvasive, and has been the most commonly used technique in children. Scoliosis is the most common abnormality of the spine with direct effects on the thoracic cage. Scoliosis has generally been associated with the development of restrictive lung disease.

Objectives: To evaluate the PFT data of children with scoliosis and to compare them to children with asthma.

**Method**: After obtaining signed informed consent from the parents we performed PFT in 50 children aged 5 to 17 years. The children were divided into four groups – 10 children with scoliosis without asthma (Sc),10 children with scoliosis and asthma (AS), 10 children with moderate asthma (BA), 10 children with severe asthma (SA) and 10 healthy children (HC).

**Results**: The four groups have similar age (p = 0.079), sex (p = 0.19) and FVC/FEV1 (p = 0.403) distribution. The results for FVC %pred, FEV1%predand MMEF 25/75%pred were significantly different with p = 0.009 (Sc vs AS), p = 0.002 (SA vs BA) and p = 0.001 (AS vs BA), all 4 groups had lower results compared to HC (p < 0.05). The same parameters were similar when comparing Sc with BA and SA with AS. The lowest FVC %pred was in the AS group, even lower than SA (p = 0.06). The children with SA demonstrated the lowest MMEF 25/75%. The FEV1% pred were higher in healthy group while children with scoliosis and asthma have comparable lower values.

**Conclusion**: The children with scoliosis demonstrated diminished expiratory flow rates, while the FEV1/FVC ratio is within normal ranges. Patients with both scoliosis and asthma, should be managed as severe asthma patients. Evaluation of their PFT is essential in their management plan for early intervention should not only restriction type deficiency is noted

**Keywords**: Scoliosis, Severe Asthma, FEV1

**Acknowledgements**: This work was supported by a grant from the Medical University of Sofia (Council of Medical Science, project no. 513/2016, grant no. 65/2016).

### P04 The cases of asthma in Japan have been expanding or not. Study on the tendency of asthma from the official statistical reports

#### Takashi Kumae

##### Research Institute, Tokyo University of Agriculture, Tokyo, Japan

**Correspondence**: Takashi Kumae - r071018@kanto-gakuin.ac.jp

*Clinical and Translational Allergy* 2017, **7(Suppl 2)**:P04

**Introduction**: For consideration and discussion of data obtained from a survey on asthma, two official statistical reports are available in Japan. The fundamental data in these reports are statistically analyzed and compared to clarify the tendency of asthma in Japan.

**Method**: One is annual report of school health statistics (SHS) from Ministry of Education, Culture, Sports, Science and Technology which has competence to survey the school age population including diseases and related health problems. The incidence of asthma of SHS from 1995 to 2015 in 5 to 17 years old are used in this report. The other is the patient survey by Ministry of health, Labour and Welfare. The patient survey covers all generations and diseases but the survey carried on every third year and numbers of patients are summarized by 5 years old. Numbers of asthma case from 1996 to 2014 are extracted and adjusted to the corresponding age population as per 1,000 capita in this report.

**Results**: According to SHS, the incidence of asthma was most frequent in 6 years old and statistically significant positive correlations were observed between the incidence and a lapse of years in all ages. Average slope of regression lines in the 5 to 9 years old was 0.10 in female and 0.16 in male. This analysis means that the incidence may be keep increase over 0.1% in annual in the near future. From the patient survey, asthma cases were most numerous in the 0 to 4 years old group and the 5 to 9 years old group was next. Asthma cases increased in elderly and the level of the 70 to 74 years old group was almost same level of the 10 to 14 years old group. Contrary to SHS, statistically significant negative correlations were observed between the asthma cases and a lapse of years in the age groups, 15 to 19 (p < 0.01), 20 to 24 (p < 0.05) and 25 to 29 (p < 0.05) years old in male. The analysis of the patient survey suggests that the asthma has decreased in these two decades.

**Conclusion**: Contradictory results in younger generation are obtained by the analysis of the two official statistical reports. The patient survey is based on actual number of persons had a medical examination in medical facilities, on the other hand, SHS is carried out using the questionnaire. The data of SHS is seemed to be influenced by the anamnesis. Especially in lower age children, SHS data are also possibly influenced by the levels of interests and anxieties for asthma in their protectors.

**Keywords**: Annual Report Of School Health Statistics, Patient Survey, Statistical Analysis

### P05 Patients’ perspectives of the impact of severe asthma on sex, intimacy and relationships: too taboo to talk about?

#### L. J. Holmes^1^, J. Yorke^2^, D. M. Ryan^3^

##### ^1^University Hospital of South Manchester & University of Manchester, Manchester, United Kingdom; ^2^University of Manchester, Manchester, United Kingdom; ^3^University Hospital of South Manchester, Manchester, United Kingdom

**Correspondence**: Leanne-Jo Holmes - leanne.holmes1@ntlworld.com

*Clinical and Translational Allergy* 2017, **7(Suppl 2)**:P05

**Introduction**: Patients with severe asthma experience unpredictable daily symptoms and an intense treatment regimen that can impact upon physical, emotional well-being and quality of life. Sexual function is a basic human requirement, contributing to quality of life; yet there is a dearth of research exploring relationships and intimacy in people with severe asthma. The overall aim was to explore the impact of severe asthma on patient’s experiences of intimacy and relationships, establish their information needs and the role of the healthcare professional.

**Method**: A qualitative study using semi-structured interviews with patients receiving treatment for severe asthma at a dedicated severe asthma clinic was undertaken. Interviews were audio recorded and transcribed verbatim. Transcripts were verified then analysed for emergent themes which were then categorised into super-ordinate themes and sub themes.

**Results**: All nine participants had a proven diagnosis of severe asthma. A higher proportion were female n = 6 (66.6%) compared to male n = 3(33.3%). The mean age was 45 years, range 34–59 years. The majority of participants 8(88.9%) were married, with 1(11.1%) co-habiting.

The analysis of interviews provided complex, detailed and novel insights into patients’ perspectives on how living with severe asthma impacts upon intimacy and spousal relationships. Four super-ordinate themes emerged:

(i) ‘Physical intimacy’ including disclosure of physical limitations of severe asthma upon intimacy and sexual activity.
(ii) ‘Emotional intimacy’ the cyclical impact of the often negative emotional struggle of living with severe asthma upon both relationships and intimacy.
(iii) ‘Image of self’ as participants divulged their battle with body image and confusion in changing relationship roles.
(iv) ‘The role of the healthcare professional’ patient identified information requirements within this area.

**Conclusion**: The participants in this study have provided invaluable and novel insights into the implications of living with severe asthma upon a sensitive and rarely discussed topic. It is anticipated that these findings will serve to increase understanding and assist practitioners to help patients to adopt positive strategies to improve their quality of life within this area.

### P06 Clinical characteristics and allergic sensitization of preschool children hospitalized with acute wheezing: a multi-cohort study

#### Sasawan Chinratanapisit^1^, Khlongtip Matchimmadamrong^2^, Jitladda Deerojanawong^3^, Wissaroot Karoonboonyanan^4^, Paskorn Sritipsukho^5^

##### ^1^Division of Allergy & Immunology, Department of Pediatrics, Bhumibol Adulyadej Hospital, Bangkok, Thailand; ^2^Saraburi hospital, Saraburi, Thailand; ^3^Division of Respiratory disease and intensive care, Department of Pediatrics, Faculty of Medicine, Chulalongkorn University, Bangkok, Thailand; ^4^Department of Pediatrics, Bhumibol Adulyadej Hospital, Bangkok, Thailand; ^5^Center of Excellence in Applied Epidemiology, Thammasat University, Bangkok, Thailand

**Correspondence**: Sasawan Chinratanapisit - sasawan2001@yahoo.com

*Clinical and Translational Allergy* 2017, **7(Suppl 2)**:P06

**Introduction**: Background: Wheezing is a significant health problem in Thailand, especially in the preschool age.

Objective: In this multi-center clinical research, clinical characteristics and biomarkers were studied to determine the factors associated with persistent wheezing in the next three years among pediatric patients with acute wheezing who were less than five years of age.

**Method**: This prospective observational study recruited children, aged 6 months to 5 years, with acute wheezing from the pediatric wards of four hospitals from July 2014 to June 2015. Patients’ characteristics and their family data were collected. Blood samples were collected for detecting ImmunoCAP specific IgE against common food and inhalant allergens. Serum 25-hydroxyvitamin D (25OHD) concentration was measured by ELISA. Spot urine samples were measured for urinary Leukotriene E4 (uLTE4) levels by enzyme immunoassay.

**Results**: Two hundred and nine data collecting forms with the bioassay results were available for analysis in this report. The mean age of the participants was 26 months with boys making up 62.5%. First wheeze episode accounted for 40.6 percent. Seventy-five percent was atopic with mono-sensitization (29.3%). The highest prevalence of sensitization was egg (45.6%), followed by cow’s milk (45.3%). The high median urine leukotriene E4 (uLTE4) levels of 226 pg/ml and 956 pg/mg creatinine was documented. The prevalence of vitamin D insufficiency, defined by the serum 25OHD levels of less than 30 ng/ml, was detected in 22.3%.

**Conclusion**: Majority of preschool children with acute wheezing admitted in the hospital had sensitization with egg and cow’s milk. One-fourth of them had vitamin D insufficiency and associated with high serum level of uLTE4.

**Keywords**: Allergic Sensitization, Acute Wheezing

### P07 Cluster analysis of patients with moderate to severe uncontrolled bronchial asthma

#### Vania Youroukova^1^, Denitsa Dimitrova^1^, Yanina Slavova^1^, Spaska Lesichkova^2^

##### ^1^Medical Faculty of Medical University of Sofia; Clinical center of pulmonary diseases; SHATPD “St. Sofia”, Sofia, Bulgaria; ^2^Medical Faculty of Medical University of Sofia, Department of Clinical Immunology, University Hospital Alexandrovska, Sofia, Bulgaria

**Correspondence**: Denitsa Dimitrova - ddimitrova87@abv.bg

*Clinical and Translational Allergy* 2017, **7(Suppl 2)**:P07

**Introduction**: The aim of the study is to identify distinct subgroups of adult patients with uncontrolled moderate to severe bronchial asthma (BA) based on clinical and inflammatory characteristics.

**Method**: Adults with uncontrolled moderate to severe asthma (n = 23) underwent a clinical assessment, spirometry and measurement of fractional exhaled nitric oxide (FeNO). Total and specific serum IgE levels, eosinophil count in peripheral blood and sputum were evaluated. K-mean cluster analysis was used to identify clinically relevant subgroups.

**Results**: We have identified four distinct groups based on onset, atopic status, FEV1/FVC ratio and FeNO. Cluster 1 patients (eosinophilic asthma) (n = 6; 26.1%) were with late-onset asthma, predominantly non-atopic with deteriorated lung function and highest level of FeNO. In this cluster were observed the highest blood and sputum eosinophil counts (p = 0.01; p = 0.04) and more frequent exacerbations (≥ 1 exacerbation in previous 12 months) (p = 0.03). Cluster 2 patients (late-onset, atopic asthma) (n = 7; 30.4%) were late-onset, mainly atopic with high total serum IgE levels, lower levels of FeNO and eosinophil counts and deteriorated lung function. Cluster 3 patients (non-atopic asthma) (n = 6; 26.1%) were late-onset, mainly non-atopic, with lower levels of FeNO and eosinophil counts and well-preserved pulmonary function. Cluster 4 patients (early-onset, atopic asthma) (n = 4; 17.4%) were early-onset, atopic, with low levels of FeNO and preserved lung function. In cluster 3 and 4 prevailed patients with high BMI (n = 7).

**Conclusion**: Our current results suggests that uncontrolled asthma is heterogeneous disease. Cluster identification will result in a personalized approach for each patient and improvement of therapy response.

The research was funded by GRANT project № 308/2015, contract №75/2015 to CMS, MU Sofia

**Keywords**: Clusters, Uncontrolled Adult Asthma, Inflammatory Characteristics

### P08 Influence of respiratory viruses on the severity of bronchial obstruction in preschool wheezing children

#### Iren Tzocheva^1^, Snezhina Lazova^1^, Snezhana Parina^1^, Svetla Angelova^2^, Neli Korsun^2^, Penka Perenovska^1^

##### ^1^Pediatric clinic, University Hospital Alexandrovska, Medical University, Sofia, Bulgaria; ^2^National Centre of Infectious and Parasitic Diseases, National Laboratory Influenza and ARD, Sofia, Bulgaria

**Correspondence**: Snezhina Lazova - snejina@lazova.com

*Clinical and Translational Allergy* 2017, **7(Suppl 2)**:P08

**Introduction**: Respiratory infections are associated with wheezing illnesses at all ages and may also impact the development and severity of asthma. Wheezing illnesses in young children are associated up to 95% with respiratory viral infections. In children with established asthma, viral respiratory tract infections play a key role in producing acute exacerbations that may lead to hospital admission. The aim of the present study is to investigate the relationship between various respiratory pathogen and severity of bronchial obstruction in hospitalized preschool children.

**Method**: Nasopharyngeal secretions of 57 children aged 2 to 5 years hospitalized with wheezing episode (Jan to Mar 2016) were examined for respiratory viruses (HMPV; RSV A/B; PIV 1, 2, 3; RV, AdV; Influenza A/B) with Real Time PCR.

**Results**: Respiratory viral infections was detected in 84% of the patients. The predominant pathogen were RSV (52%), followed by influenza virus A (25%), human metapneumovirus (12.5%), rhinovirus (4%), parainfluenza (4%) and adenovirus (2.5%). In children with life-threatening bronchial obstruction with a need of prolonged oxygen therapy, RSV was the most commonly detected pathogen (42%). The presence of viral infection did not correlate with the duration of the systemic corticosteroid treatment. The median duration of reported lung sounds (wheezing and crackles) was longer in the patients with detected virus infection without statistical significance (p = 0.06).

**Conclusion**: Respiratory viruses play a key role in the wheezing illnesses in young children, predominantly infants. Our results demonstrated that the RSV is an important pathogen in severe, life-threatening episodes in preschool age children as well. Contrary to data presented in literature, human rhinovirus was detected in only 4% of our patients following Influenza A and human metapneumovirus.

**Keywords**: Preschool Wheezing, Respiratory Viruses, Asthma Exacerbation, Severity

### P09 Tele-monitoring decreases unscheduled outpatient visits in pediatric patients with severe asthma

#### Mihai Craiu, Iustina Violeta Stan

##### INSMC Alessandrescu-Rusescu, Bucharest, Romania

**Correspondence**: Mihai Craiu - mcraiu@yahoo.com

*Clinical and Translational Allergy* 2017, **7(Suppl 2)**:P09

**Introduction**: Pediatric asthma patients originating from poor-resource areas experience a significant increase of unscheduled and emergency department visits and most of medical care for them is delivered in such a facility. Education and communication is vital for severe asthma patients. Virtual media can be used to improve asthma control in these patients. Official data are documenting that ¾ of citizens own at least a smart-phone in our country and even in poor areas there is excellent mobile phone coverage. The aim of our study was to document outcome of a structured tele-monitoring approach (via virtual media) in management of severe asthmatic children.

**Method**: Prospective 12 months study of patients with severe asthma. Were observed severe asthma patients from a cohort of previously monitored children with uncontrolled asthma [ACT child score below 19] and frequent visits in Emergency Department in previous year. Extensive education in basic inhalation techniques [video-training] was done and written-action plan (WAP) was provided. Were trained to use PIS [Pulmonary Index Score] to evaluate severity of exacerbation and treatment response. Parents had to either use ED or use WAP and tele-monitoring. Short video of clinical status was evaluated by an expert and further options were discussed. Non-responders were presented in ED.

**Results**: In 2014 were 880 ED visits of 296 patients. 16 children were included in the tele-monitoring study, 4 with severe asthma and 38 as controls. Patients had male predominance [75% vs 71.1% in controls]. 2 (1 of the 4 severe asthma patients) vs 11 children in control group had more than 1 visit to ED in 2014. Odds-ratio to have an unscheduled ED visit was 0.1964 in tele-monitoring group [p 0.0483, 95% CI 0.039 – 0.9881]. Was higher for severe asthma patients compared with non-severe with an odds–ratio 3.667 but non-significant [p 0.4040, 95% CI 0.1734 to 77.5559]

**Conclusion**: Tele-monitoring via virtual media can decrease the burden of unscheduled ED visits for asthmatic children, even in severe asthma patients.

**Keywords**: Child, Asthma, Virtual-Media, Monitoring, Education

### P10 Challenges in using hierarchical clustering to identify asthma subtypes: choosing the variables and variable transformation

#### Matea Deliu^1^, Tolga Yavuz^2^, Matthew Sperrin^3^, Umit M. Sahiner^4^, Danielle Belgrave^5^, Cansin Sackesen Sackesen^6^, Adnan Custovic^7^, Ömer Kalayci^8^

##### ^1^Institute of Population Health, Health e-Research Centre, University of Manchester, Manchester, United Kingdom; ^2^Division of Pediatric Allergy, GATA Military School of Medicine, Ankara, Turkey; ^3^Centre for Health Informatics, Institute of Population Health, University of Manchester, Manchester, United Kingdom; ^4^Division of Pediatric Allergy, Hacettepe University School of Medicine, Ankara, Turkey; ^5^Faculty of Medicine, Department of Medicine, Imperial College London, London, United Kingdom; ^6^Koç University Hospital, Istanbul, Turkey; ^7^Department of Paediatrics, Imperial College London, London, United Kingdom; ^8^Pediatric Allergy and Asthma Unit, Hacettepe University School of Medicine, Ankara, Turkey

**Correspondence**: Matea Deliu - matea.deliu-2@postgrad.manchester.ac.uk

*Clinical and Translational Allergy* 2017, **7(Suppl 2)**:P10

**Introduction**: The use of unsupervised clustering has identified different subtypes of asthma. Choosing the variables to input into the clustering algorithm is one of the important considerations. The majority of previous studies selected variables based on expert advice, whilst others used dimension reduction techniques such as principal component analysis (PCA). We aimed to compare the results of unsupervised clustering when using raw variables, or variables transformed using dimensionality reduction techniques.

**Method**: We performed our analysis on 613 asthmatics aged 6–23 years from Ankara, Turkey. We conducted extensive phenotyping and recorded 49 variables including demographic data, sensitization, lung function, medication, peripheral eosinophilia, and markers of asthma severity. We performed hierarchical clustering (HC) using: (1) all variables; and (2) variables transformed using dimensionality reduction techniques.

**Results**: PCA revealed 5 components describing atopy and variations in asthma severity, which were then used to infer cluster assignment. The optimal HC solution in both PCA-transformed and raw untransformed data identified five clusters. However, these clusters were not identical. Both identified mild asthma with good lung function, severe atopic asthma and late-onset mild atopic asthma. However, the overlap between children assigned to these three clusters in two HC analyses was modest. Clustering without PCA identified early-onset severe atopic asthma and late-onset atopic asthma with high BMI, whilst early onset non-atopic mild asthma in females was identified in HC with PCA.

**Conclusion**: Different methodologies applied to the same dataset identified differing clusters of asthma. Despite cluster instability, both methodologies provided meaningful clinical insights for understanding asthma heterogeneity.

## POSTER DISCUSSION SESSION 1—Biomarkers

### P11 Th17 in children with asthma

#### Snezhina Lazova^1^, Penka Perenovska^1^, Guergana Petrova^1^, Dimitrinka Miteva^1^, Petar Velikov^2^, Tsvetelina Velikova^3^, Ekaterina Ivanova-Todorova^3^, Kalina Tumangelova-Yuzeir^3^, Dobroslav Kyurkchiev^3^

##### ^1^Medical University, Pediatric clinic, UMHAT Alexandrovska, Sofia, Bulgaria; ^2^Medical University, Sofia, Bulgaria; ^3^Medical University, Laboratory of Clinical Immunology, University Hospital St. Ivan Rilski, Sofia, Bulgaria

**Correspondence**: Snezhina Lazova - snejina@lazova.com

*Clinical and Translational Allergy* 2017, **7(Suppl 2)**:P11

**Introduction**: Th17 lymphocytes are now widely believed to be critical for the regulation of various chronic immune diseases, including asthma and chronic obstructive pulmonary disease.

Objectives: To assess Th17 levels in children with asthma (BA) and cystic fibrosis (CF).

**Method**: We included 12 children with CF without history of allergies and 20 children with BA aged 7 to 16 years (10 with severe BA and 10 with moderate BA). After informed consent, 1.2 ml of venous blood was collected during a routinely performed blood withdrawal. Analyses of Th17 cells were performed with Lise-Wash Protocol by a 4-color FacsCalibur flow cytometer after staining the whole blood with the following fluorescence-labeled antibodies: anti-CD3 (FITC), anti-CD (PerCP), anti-CD161 (PE) and anti-CCR6 (AlexaFluor 647).

**Results**: The patients in asthma group had significantly higher percentage of Th17 12.40% ± 1.16 compared to the children with CF - 7.64 ± 0.87 (p = 0.008). Stratifying the BA group according the severity we found higher levels of Th17 in patients with severe BA (p < 0.05). Patients with moderate asthma had TH17 values close to those in CF children.

**Conclusion**: The number of Th17 cells is significantly increased in the peripheral blood of children with severe BA compared to the children with moderate BA. Severe BA patients could have possible benefit from the future target therapies.

**Keywords**: Severe Asthma, Controlled Asthma, Cystic Fibrosis

**Acknowledgements**: This work was supported by a grant from the Medical University of Sofia (Council of Medical Science, project no. 289/2015, grant no. 54/2015).

### P12 Study of the viral and bacterial communities associated with asthma: a metagenomic approach

#### Spyridon Megremis^1^, Bede Constantinides^1^, Alexandros Georgios Sotiropoulos^1^, Paraskevi Xepapadaki^2^, David Robertson^1^, Nikolaos Papadopoulos^1^

##### ^1^University of Manchester, Manchester, United Kingdom; ^2^University of Athens, Athens, Greece

**Correspondence**: Spyridon Megremis - spyridon.megremis@manchester.ac.uk

*Clinical and Translational Allergy* 2017, **7(Suppl 2)**:P12

**Introduction**: An important unexplored source of interpersonal variation in asthma presentation is the complex and dynamic community of microorganisms located on different sites of the respiratory tract. Our aim was to identify and explore the bacterial and viral diversity in a small pilot study of asthmatic children using metagenomics.

**Method**: Nasopharyngeal samples were obtained from five asthmatic children (2 males) at a baseline asymptomatic visit. Mean age was 5.2 years (range 4.6–5.8). Communities of viruses (DNA and RNA genomes) and bacteria were evaluated using high depth paired-end shotgun sequencing. We performed taxonomic classification of sequences and calculated environmental indexes (alpha and beta) to estimate the microbial diversity both within (Shannon, Chao1, Jack1) and between (Jaccard) patients. Rarefaction curves were produced to study the relationship between microbial diversity and the number of generated sequences and/or number of samples. A correlation matrix was used to identify positive and negative correlations between bacterial and viral families and the results were superimposed in a network graph.

**Results**: Taxon classification revealed 198 bacterial and 20 viral families. Unsupervised hierarchical clustering based on beta diversity (Jaccard matrix) clustered controlled, partly controlled and uncontrolled asthma separately. Topographical analysis of the metagenomic interaction map identified the phage family of Myoviridae as a central component. Further analysis of the Myoviridae family and its direct interactions with bacterial families (17 families) revealed that patients with partly controlled and uncontrolled asthma have reduced quantity of Myoviridae phages and reduced diversity and quantity of their bacterial interactors.

**Conclusion**: Based on the Rarefaction curves, we estimate that, even with 5 samples a high percentage of the microbial diversity can be robustly represented. Asthma patients were efficiently clustered based on their microbial similarity which seems to be representative of asthma control. The phage family Myoviridae is predicted to hold a central role in the viral-bacterial interaction on the specific environmental site. The reduced number of reads of the Myoviridae phages along with the reduced diversity and quantity of 17 bacterial families in uncontrolled asthma could be evident of an ongoing ‘fight’ between components of the upper respiratory metagenome.

**Keywords**: Asthma, Metagenomics, Respiratory, Children

### P13 Circadian breathomics in asthma: analysis by thermal desorption-gas chromatography-mass spectrometry

#### Maxim Wilkinson^1^, Craig Portsmouth^1^, David Ray^2^, Royston Goodacre^3^, Stephen J. Fowler^4^, Hannah Durrington^2^

##### ^1^Division of Infection, Immunity and Respiratory Medicine, Faculty of Biology, Medicine and Health, University of Manchester, Manchester, United Kingdom; ^2^Division of Metabolism and Gastroenterology, Faculty of Biology, Medicine and Health, University of Manchester, Manchester, United Kingdom; ^3^School of Chemistry, Manchester Institute of Biotechnology, University of Manchester, Manchester, United Kingdom; ^4^Manchester Academic Health Science Centre, and NIHR Respiratory and Allergy Clinical Research Facility, University of Manchester, University Hospital of South Manchester, Manchester, United Kingdom

**Correspondence**: Maxim Wilkinson - maxim.wilkinson@postgrad.manchester.ac.uk

*Clinical and Translational Allergy* 2017, **7(Suppl 2)**:P13

**Introduction**: Asthma is closely associated with the endogenous circadian rhythm of the lungs; in severe and uncontrolled asthma nocturnal wakening is common, as is a dip in early morning peak expiratory flow. This variability makes asthma a potential target for chronotherapy to improve the efficacy of treatment. To personalise our approach to chronotherapy in asthma, it is essential to have an easy-to-measure biomarker that reflects an individual patient’s chronotype. This would allow chronotherapy to be targeted to the right time of day for each patient. Breath samples are easy to collect and non-invasive, and exhaled volatile organic compounds (VOCs) have been related to airway inflammatory patterns in asthma. We hypothesise that the exhaled VOC profile contains potential biomarkers that track the circadian rhythm in asthma. Samples are analysed using thermal desorption-gas chromatography–mass spectrometry which is currently the gold standard for offline VOC detection, and allows high sensitivity and reliable identification of detected compounds.

**Method**: Twenty non-smoking volunteers (10 healthy, 10 with moderate asthma) will be recruited. So far nine individuals have been recruited; the study design is detailed in the figure. Breath samples are taken at 15:00 on the first visit; 15:00, 21:00, 03:00 and 09:00 on the second and 09:00 on the final visit. This gives six samples per participant at four time points allowing investigation of both the changes in an individual’s VOC profiles over the course of a day and longitudinal variation over the course of several weeks. All samples are analysed using thermal desorption-gas chromatography-mass spectrometry

**Results**: The extracted ion chromatograms for the internal standard (p-bromofluorobenzene), isoprene, limonene and the alkanes are shown as a quality control procedure to ensure clean data processing. Preliminary results will be presented by multivariate analysis for the first nine participants comprising six individuals with asthma and three controls.

**Conclusion**: Measuring exhaled breath VOCs at intervals throughout the day may provide a useful way of mapping a patient’s asthma chronotype, guiding future chronotherapy.

**Keywords**: Circadian Rhythm, TD-GC–MS, Breathomics

**Figure:**
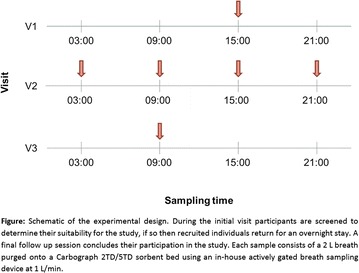
Figure 4 Schematic of the experimental design

### P14 Serum periostin levels as a biomarker for impaired lung function in adult patients with moderate to severe asthma

#### Denitsa Dimitrova^1^, Vania Youroukova^1^, Tsvetelina Velikova^2^, Kalina Tumangelova-Yuzeir^2^, Anna Valerieva^3^

##### ^1^Medical Faculty of Medical University of Sofia; Clinical center of pulmonary diseases; SHATPD “St. Sofia”, Sofia, Bulgaria; ^2^Medical Faculty of Medical University of Sofia, Department of Clinical Laboratory and Clinical Immunology, University Hospital, Sofia, Bulgaria; ^3^Medical Faculty of Medical University of Sofia, Clinic of Allergy and Asthma, University Hospital Alexandrovska, Sofia, Bulgaria

**Correspondence**: Denitsa Dimitrova - ddimitrova87@abv.bg

*Clinical and Translational Allergy* 2017, **7(Suppl 2)**:P14

**Introduction**: The aim of the study is to determine the relationship between serum periostin (SP) levels and clinical characteristics in adult patients with moderate to severe asthma (AP).

**Method**: SP levels were measured by ELISA method in 42 AP out of exacerbations and 10 healthy controls (HC). AP were divided into two groups according asthma control test (ACT) score: controlled AP (CA, n = 19) and uncontrolled AP (UA, n = 23). Atopic status was confirmed by skin prick testing and measurement of total and specific serum IgE levels. Lung functional parameters were assessed by spirometry.

**Results**: We have observed significantly higher levels of SP in AP (1.68 ± 0.52 ng/ml) compared to HC (1.08 ± 0.12 ng/ml) (p < 0.001). No statistically significant differences were found in SP levels between CA and UA, atopic (n = 14) and non-atopic AP (n = 28), current smokers (n = 13) and non-smokers (n = 29) (p = 0.85, p = 0.33, p = 0.19 respectively). We have found significantly higher SP levels in AP on medium (n = 22) and high doses ICS (n = 9) compared to those on low dose ICS (n = 11) therapy (p = 0.011, p = 0.007 respectively). Twenty AP (n = 5, CA and n = 15, UA) demonstrated impaired lung function (FEV_1_ < 80%), which was associated with higher levels of SP (p = 0.04). Similarly, in UA was observed significantly negative correlation between SP and FEV_1_ % (p = 0.006, r = − 0.56).

**Conclusion**: Patients with persistent moderate to severe bronchial asthma show higher levels of SP as compared to healthy controls. High levels of SP are associated with patient need of higher dose ICS therapy. Our results suggest that SP is biomarker for impaired lung function in poor asthma control.

**Keywords**: Periostin, Severe Adult Asthma, Lung Function

The research was funded by GRANT project No. 308/2015, contract No. 75/2015 to CMS, MU Sofia

### P15 Serum periostin values in healthy non-asthma non-copd spanish adults

#### Irina Bobolea, Daiana Guillén Vera, Gabriel Gonzalez-Salazar, Carlos Melero Moreno, Consuelo Fernandez Rodriguez, Natividad De Las Cuevas Moreno

##### Hospital 12 de octubre, i + 12 Research Institute, Madrid, Spain

**Correspondence**: Irina Bobolea - ibobolea@gmail.com

*Clinical and Translational Allergy* 2017, **7(Suppl 2)**:P15

**Introduction**: Serum periostin is an emerging biomarker for T2 asthma, and for the response to new biologicals such as anti IL-13 lebrikizumab. But a clear cut-off has not been established, and other pathologies beyond asthma are known to course with high periostin levels. The aim of this study was to establish reference values for serum periostin in a representative sample of non-asthmatic non-COPD healthy population in our geographical area.

**Method**: A cross-sectional study was conducted between May–October 2015. After signing the informed consent, we collected sera samples from consecutive blood donors of the Blood Bank of Universitary Hospital 12 de Octubre, Madrid, Spain.

By choosing this source of donors, all diseases and other conditions related to any periostin elevations described so far were excluded, as they constitute contraindications for donating blood: chronic illnesses (diabetes; heart, pulmonary, renal or hepatic diseases); any type of cancer; chronic or acute infectious diseases, including stomathologycal disordes; pregnancy; recent traumatic injury or surgery. Moreover, all participants completed a questionnaire aimed to exclude those with symptoms and/or a physician diagnosis of asthma and/or COPD. Sera were frozen at −80 °C until periostin was measured using an EISA commercial kit. We calculated the sample size to estimate a media, using a standard deviation (SD) of 15, with 3 ng/mL precision and 95% confidence interval (*Anastasilakis* et al*. 2014*).

**Results**: 100 non-asthmatic non-COPD and otherwise healthy subjects, aged between 18 and 65 years, were included in the study. Results are expressed in media (SD) (min–max range). Age: 43.97 years (10.35) (21–65 years). 62 males (62%). 22 smokers (25.9%); being the official percentage of smokers in the Madrid area 27.4%.

Serum periostin: 33.04 ng/mL (14.37) (6.87–81.43 ng/mL). The media estimated in total population was (30.19–35.90 ng/mL) with a confidence interval of 95%. Periostin in males: 33.51 ng/mL (14.02), and females: 32.28 ng/mL (15.09), p = 0.680. Smokers: 32.4 ng/mL (12.05) and non-smokers: 33.09 ng/mL (15.28) respectively, p = 0.850. No association was either found between age and serum periostin (r = 0.075).

**Conclusion**: We report reference values for serum periostin in a healthy non-asthmatic non-COPD adult Spanish population. Our results could be used as reference for further studies aimed to clarify the role of periostin in asthma phenotypes and endotypes, or to choose future candidates for the new biological agents targeting T2 inflammation.

**Keywords**: Asthma Biomarkers, Periostin

## POSTER DISCUSSION SESSION 2—Mechanisms

### P18 ToAST: Investigating the effects of bronchial thermoplasty (bt) on cough responses in patients with severe asthma – a pilot study

#### R. Wang, I. Satia, R. Niven, S. J. Fowler, D. M. Ryan, J. A. Smith

##### University Hospital of South Manchester, Manchester, United Kingdom

**Correspondence**: Ran Wang - ranwang1986@googlemail.com

*Clinical and Translational Allergy* 2017, **7(Suppl 2)**:P18

**Introduction**: Severe asthma affects 5% of the asthma population but drives the majority of the morbidity and cost. Despite cough being a major symptom and a marker of both severity and poor prognosis, current medications are not designed to treat cough directly. Bronchial thermoplasty (BT) is a novel therapy in severe asthma, delivering radiofrequency thermal energy to the large airways. There are provisional data suggesting BT reduces the number of airway nerves^1^. If this is the case, then BT may affect cough in asthma. Inhaled capsaicin challenge has been safely used to evoke cough responses and to assess cough threshold in research. However, to date no study has adopted this method to assess cough in patients with severe asthma or following BT.

We aim to assess the safety and feasibility of capsaicin challenge in people with severe asthma. We will also explore the effects of BT on 24 h cough frequency, capsaicin evoked cough responses and cough-related quality of life.

**Method**: The protocol for this two-visit observational study has been approved by the local Research Ethic Committee. All recruited patients (target n = 24, of whom half will have had BT) will be treated at British Thoracic Society steps 4 or 5. During visit 1 patients will be consented, the Leicester Cough and Asthma Control Questionnaires administered and baseline spirometry performed. They will undergo ambulatory 24-hour cough monitoring. A capsaicin challenge will be performed during the second visit. Spirometry will be performed before and immediately after the challenge to assess any change in FEV_1_.

**Results**: The primary outcome measure will be the number of participants with a 20% or greater fall in FEV1 and/or requiring rescue bronchodilator therapy during the capsaicin challenge period. Secondary outcomes include: change in  %FEV1 predicted during capsaicin challenge; tolerability of inhaled capsaicin; number of adverse events during and after capsaicin evoked cough challenge; differences in capsaicin dose response curves between patients who have had BT versus those have not.

**Conclusion**: This pilot study will lead to a larger prospective study investigating the effects of BT on cough responses in patients with severe asthma.

**Reference**

1. Bergqvist A, Pretolani M, Taille C, Dombret MC, Knapp D, Bjermer L, Chanez P, Erjefalt JS, Aubier M. Selective structural changes of bronchial thermoplasty in the treatment of severe uncontrolled asthma. Am J Respir Crit Care Med. 2015;191:A4171.

### P19 Anti-inflammatory capability of Phosphatidylinositol 3-kinase delta inhibitors in moderate to severe asthma patients

#### T. Southworth^1^, J. Plumb^1^, V. Gupta^1^, J. Pearson^1^, I. Ramis^2^, M. D. Lehner^2^, M. Miralpeix^2^, D. Singh^1^

##### ^1^The University of Manchester, Manchester, United Kingdom; ^2^Almirall S. A., Barcelona, Spain

**Correspondence**: Thomas Southworth - tsouthworth@meu.org.uk

*Clinical and Translational Allergy* 2017, **7(Suppl 2)**:P19

**Introduction**: Lymphocytes numbers are increased in the lungs of asthma patients, with T-helper 2 cells being associated with eosinophilic inflammation. There is also growing evidence that T-helper 1 and T-helper 17 cells may play a role in more severe asthma. Inhaled corticosteroids (ICS) are widely used anti-inflammatory treatments for asthma, but many moderate to severe asthma patients suffer from persistent symptoms, despite using high doses of ICS. Phosphatidylinositol 3-kinase delta (PI3 Kδ) is predominantly expressed in inflammatory cells and is involved in lymphocyte activation, proliferation and differentiation. Targeting PI3 Kδ may have therapeutic benefit in currently uncontrolled asthma patients.

The aims of the study were to compare the expression PI3 Kδ in airway samples from asthma patients and healthy subjects and to assess the anti-inflammatory potential of PI3 Kδ inhibitors on bronchoalveolar lavage (BAL) lymphocytes.

**Method**: Bronchial biopsies, BAL cells and blood cells were collected from moderate to severe asthma patients and healthy subjects. The numbers of PI3 Kδ-positive cells in biopsies was calculated using immunohistochemistry and the expression of PI3 Kδ in BAL CD3 cells was assessed by flow cytometry. BAL and blood cells were treated with a PI3 Kδ inhibitor and/or dexamethasone prior to T cell receptor (TCR) stimulation. ELISAs were used to assess levels of IFNγ, IL-13 and IL-17 and flow cytometry was used to measure pSTAT5, an early marker of T-cell activation.

**Results**: Bronchial biopsies from asthma patients contained increased numbers of PI3 Kδ-positive cells compared to those from healthy subjects, and asthma BAL T-cells expressed higher levels of PI3 Kδ than healthy cells. Inhibition of PI3 Kδ reduced TCR-stimulated IFNγ, IL-13 and IL-17 in BAL cells from both asthma patients and healthy subjects, and treating BAL cells with a combination of PI3 Kδ inhibitor and dexamethasone had an additive effect on cytokine inhibition. TCR-induced pSTAT5 in purified T-cells and BAL cells was impeded by PI3 Kδ inhibition and dexamethasone.

**Conclusion**: Inhibition of PI3 Kδ in asthma may be an effective method of reducing activation of T-cells and subsequent cytokine production.

**Keywords**: Phosphatidylinositol 3-Kinase Delta, Airway T-Cells, Bronchoscopy, Corticosteroids, Cytokines

### P20 Investigating neuronal responses by assessing capsaicin evoked cough responses in an allergen challenge model of asthma (INCA)

#### Imran Satia^1^, Stephen Fowler^1^, Mark Woodhead^1^, Paul O’Byrne^2^, Jaclyn Ann Smith^1^

##### ^1^University of Manchester, Manchester, United Kingdom; ^2^McMaster University, Hamilton, Canada

**Correspondence**: Imran Satia - imran.satia@manchester.ac.uk

*Clinical and Translational Allergy* 2017, **7(Suppl 2)**:P20

**Introduction**: Cough is a common and troublesome symptom in asthma but little is known about the neuronal pathways that trigger cough. The mechanisms by which airway inflammation, bronchial hyper-responsiveness and variable airflow obstruction cause cough is unclear. We have previously shown that methacholine induced bronchoconstriction heightens cough responses to inhaled capsaicin. The aim of the current study was to investigate the effects of allergen exposure on cough reflex sensitivity.

**Method**: We performed a 8 visit randomised, single-blind, placebo controlled, two-way cross-over study comparing cough responses to inhaled capsaicin in allergic asthma patients during and 24 h after exposure to allergen compared with diluent (saline) control.

Mild atopic asthmatics who are dual responders will undergo full dose allergen/diluent (saline) challenge. Early asthmatic responses (EAR) (fall in FEV1 > 20% at 30 min) and late asthmatic responses (LAR) (fall in FEV1 > 15% at 3–7 h) will be documented. Full dose capsaicin challenge was performed at screening to determine the excitatory dose evoking half the maximum cough response (ED50). The ED50 concentration of capsaicin was inhaled 4 times, 30 s apart, at 30 min and 24 h after inhaled allergen/diluent challenge. Cough counts will be measured throughout, along with methacholine challenge and induced sputum before and after allergen challenge.

**Results**: We report interim analysis of the first 5 completed patients recruited to this study (mean age (SD) 23.4 yrs (4.6), BMI 23.9 (4.3), FEV1% predicted 97.4% (8.0), PC20 1.55 (1.72), 2 female). All patients were steroid naive and sensitive to house dust mites. Mean fall in %FEV1 from baseline in the EAR was 34.9% (SD6.4), and LAR 29.2% (20.6). Baseline ED50 concentration was 24.2microM(23.8). There was an increase in capsaicin evoked coughs after allergen exposure compared to diluent at both 30 min and 24 h (mean coughs after allergen vs. diluent at 30 min 15.0 (4.9) vs. 9.6(3.5), at 24 h 12.2 (8.0) vs. 6 (2.6). Figure 1

**Conclusion**: We have safely performed capsaicin challenge after allergen exposure. Initial data suggests cough responses may be heightened for up to 24 h after allergen exposure.

Our results show the importance of STAT6 and the possible implication of SOCS proteins in the enhanced CCL26 expression by BECs from severe eosinophilic asthmatics (Supported by the Fondation de l’IUCPQ and the JD-Bégin Research Chair).

**Keywords**: Asthma, Cough, Capsaicin, Allergen Challenge, TRPV1

**Figure:**
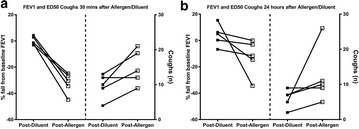
Figure 5 FEV1 and ED 50 Coughs 30 min and 24 h after Allergen/Diluent

### P21 Mechanisms of cross-talk between pulmonary epithelial cells and dendritic cells during type 2 inflammation

#### Cecilia Forss^1^, Peter Cook^1^, Sheila Brown^1^, Freya Svedberg^1^, Katherine Stephenson^1^, Margherita Bertuzzi^2^, Elaine Bignell^2^, Malin Enerbäck^3^, Danen Cunoosamy^3^, Andrew Macdonald^1^

##### ^1^Manchester Collaborative Centre for Inflammation Research, Manchester, United Kingdom; ^2^Manchester Fungal Infection Group, Manchester, United Kingdom; ^3^AstraZeneca R&D, Mölndal, Sweden

**Correspondence**: Cecilia Forss - cecilia.forss@postgrad.manchester.ac.uk

*Clinical and Translational Allergy* 2017, **7(Suppl 2)**:P21

**Introduction**: Everyday exposure to potentially harmful pathogens is carefully monitored and controlled by the interaction between a number of different cell types, such as epithelial cells and dendritic cells (DCs). Both have the ability to sense environmental changes, and initiate and direct an immune response. This project is focusing on characterizing the interactions between epithelial cells and DCs in the early stages of an allergen exposure. *Aspergillus fumigatus* (*Af*) is an opportunistic fungus with the capacity to cause a range of responses, from low-level inflammation in healthy people to life-threatening conditions in the immunocompromised.

**Method**: We have concentrated on understanding early events in the immune response towards *Af*, with a current emphasis on DCs.

**Results**: 4 h post exposure, ~ 50% of in vitro generated DCs were positive for *Af* spores and the majority of the spores were internalised. *Af* positive DCs showed higher expression of CD40, CD80 and CD86 compared to cells that were spore negative. Interestingly, while *Af* induced intermediate up-regulation of DC activation markers, it induced only low levels of cytokine production. As the type of interaction between DCs and *Af* likely influences downstream activation and polarization of T cells, we are currently dissecting the impact of internalised vs. surface bound spores, compared to soluble fungal extracts, on activation of DCs in vitro. In parallel, we have used murine GM-CSF- and Flt3-L-derived DCs and human monocyte derived DCs, as well as human bronchial epithelial cells (HBECs) in air–liquid interface (ALI) cultures. Additionally, we have used a murine model of intranasal exposure to low doses of live *Af* to investigate *Af* uptake by, and activation of, pulmonary DC subsets and epithelial cells in vivo.

**Conclusion**: Data generated so far indicate a fungal life stage-dependent up-regulation of activation markers, accompanied by induction of low-level secretion of inflammatory cytokines. *Af* exposure in vivo resulted in marked airway inflammation with infiltration of immune cells, goblet hyperplasia and increased collagen deposition. Ongoing work is addressing the ability of *Af* activated DCs to influence T cell activation and polarization in vitro and in vivo.

**Keywords**: Dendritic Cells, Bronchial Epithelial Cells, Aspergillus Fumigatus

### P22 Flavonoid cyanidin acts through IL-17RA to alleviate IL-17A-mediated severe asthma

#### Caini Liu, Liang Zhu, Kiochi Fukuda, Cunjin Zhang, Suidong Ouyang, Xing Chen, Luke Qin, Suguna Rachakonda, Mark Aronica, Jun Qin, Xiaoxia Li

##### Cleveland Clinic, Cleveland, United States

**Correspondence**: Caini Liu - liuc@ccf.org

*Clinical and Translational Allergy* 2017, **7(Suppl 2)**:P22

**Introduction**: Interleukin (IL)-17A plays a critical role in the pathogenesis of asthma. High levels of IL-17A are found in bronchial biopsies and serum obtained from patients with severe asthma. Deficiency of IL-17A signaling components attenuated airway inflammation in several mouse models of asthma. We recently identified a potent small molecule compound A18 (called cyanidin, a key pigment present in red berries and other fruits) for IL-17A inhibition through computer-aided docking-based virtual screening. A18 specifically binds to a region in the IL-17RA extracellular domain that overlaps with the binding site for IL-17A and this binding potently disrupts the IL-17A/IL-17RA complex. We demonstrated that cyanidin effectively inhibited IL-17A-mediated inflammatory gene expression in both human and mouse cells. In the current study, we further investigated if A18 could attenuated airway inflammation in mouse models of steroid-resistant and severe asthma.

**Method**: **Adoptive transfer models for antigen-induced airway inflammation**

OVA-specific Th17 and Th2 cells were transferred intravenously (i.v.) into WT C57BL/6 female mice. Mice were challenged (i.n.) with OVA_323–339_ 1 day before transfer and 3 consecutive days after transfer. For A18 treatment, mice were injected (i.p.) with A18 on each challenge day. Measurements were performed 24 h after the last challenge.

**High-fat-diet (HFD) intervention**

Starting at 4 weeks of age, WT C57BL/6 male mice were fed with 10 kcal  % fat chow diet (CD) or a 60 kcal  % HFD for 14 weeks. A18 group of mice were injected (i.p.) with A18 daily for 4 weeks before subjection to measurements.

**House dust mite (HDM)-induced asthma**

WT C57BL/6 female mice were sensitized (s.c.) with HDM (100 µg/mouse) in Complete Freund’s Adjuvant on day 0 and subsequently challenged (i.n.) with HDM (100 µg/mouse) on day 14. A18 group of mice were injected (i.p.) with A18 on days 13 and 14 and also administrated (i.n.) with A18 1 h before challenging. Measurements were performed 24 h after the last challenge.

**Results**:

A18 inhibited Th17-induced but not Th2-induced airway inflammation.

A18 substantially attenuated steroid-resistant obesity-associated asthma.

A18 alleviated House dust mite (HDM)-induced neutrophilic airway inflammation.

**Conclusion**: The findings strongly suggest that A18 (cyanidin) can be used as the prototype for developing small molecule drugs for treating the IL-17A-mediated severe asthma.

**Keywords**: Cyanidin, IL-17A, Severe Asthma

### P23 Mechanisms involvement in CCL26 production by bronchial epithelial cells: importance in asthma and its severity

#### Marie-Chantal Larose, Anne-Sophie Archambault, Véronique Provost, Jamila Chakir, Michel Laviolette, Nicolas Flamand

##### CRIUCPQ, Québec, Canada

**Correspondence**: Marie-Chantal Larose - marie-chantal.larose@criucpq.ulaval.ca

*Clinical and Translational Allergy* 2017, **7(Suppl 2)**:P23

**Introduction**: High pulmonary eosinophil counts correlate with asthma severity and exacerbation. We recently showed that sputum CCL26 levels correlate with sputum eosinophils. We also found that among all CC chemokines, IL-13 selectively induced the expression of CCL26 by bronchial epithelial cells (BECs) and this phenomenon is significantly enhanced in BECs from severe eosinophilic asthmatics. We postulated that the superior CCL26 production that we observed in severe asthmatics was the consequence of increased signaling events mediated by IL-13. We thus assessed the expression and functional responses of the different signaling effectors linked to the IL-13 signaling in CCL26 expression by BECs from healthy subjects, mild asthmatics, and severe eosinophilic asthmatics.

**Method**: Human primary BECs were isolated and cultured from bronchial biopsies. BECs were treated with IL-13 or vehicle for different times. Inhibitors or their vehicles were added to BECs 1 h before IL-13. CCL26 expression was assessed by qPCR and ELISA. STAT6 and phosphorylated (p)STAT6 were quantitated by ELISA in BEC lysates. SOCS3 level were assessed by immunoblotting.

**Results**: We confirmed the involvement of STAT6 in the induction of CCL26 expression by treating BECs with increasing STAT6 inhibitor. This led to a concentration-dependent inhibition of CCL26 expression in IL-13-stimulated BECs. In that regard, the pSTAT6/STAT6 ratios were increased in IL-13-stimulated BECs from severe eosinophilic asthmatics, compared to those from healthy subjects and mild asthmatics. This increased activation of STAT6 might be explained by decreased SOCS3 level in BECs from severe eosinophilic asthmatics. Also, we confirmed the importance of STAT6 and IL-13 in CCL26 expression and production in IL-13-treated-BECs throughout the incubation time.

**Keywords**: CCL26, Eosinophils, Bronchial Epithelial Cells

### P24 Chitinase-like proteins: the missing link in allergen-induced neutrophilic inflammation?

#### Nicola Logan^1^, Dominik Ruckerl^2^, Judith E. Allen^2^, Tara E. Sutherland^3^

##### ^1^Institute of Immunology and Infection Research, University of Edinburgh, Edinburgh, United Kingdom; ^2^Faculty of Biology, Medicine & Health, University of Manchester, Manchester, United Kingdom; ^3^Manchester Collaborative Centre for Inflammation Research, University of Manchester, Manchester, United Kingdom

**Correspondence**: Tara E. Sutherland - tara.sutherland@manchester.ac.uk

*Clinical and Translational Allergy* 2017, **7(Suppl 2)**:P24

**Introduction**: Immune responses during asthma are typically considered as T-helper 2 (Th2) type responses marked by increased eosinophils. However, inflammation in severe asthmatics is often defined by neutrophil or mixed neutrophil/eosinophil airway inflammation, along with increased IL-17 cytokine levels and airway remodelling. Little is known about the triggers that make neutrophils/IL-17 dominant characteristics in severe asthmatics, or how these responses contribute to pathology, especially in scenarios when Th2-inflammation remains prevalent. Chitinase-like proteins (CLPs) are established markers of immune activation and pathology. Of note, whilst considered Th2-driven molecules, CLPs are also associated with IL-17 and neutrophilic inflammation. In asthmatic patients, enhanced levels of circulating CLP YKL-40 correlates with disease severity and airway neutrophilia^1^. In addition, we recently described a novel role for murine CLPs in driving alveolar neutrophil recruitment through increased IL-1/IL-18 expression and expansion of IL-17A-producing γδ T cells^2^. Considering their association with both Th2 pathology and IL-17A/neutrophils, we sought to examine whether CLPs might link IL-17 responses to Th2 inflammation.

**Method**: We explored the effects of neutralising the most abundant murine CLP Ym1, with monoclonal antibody treatment during infection with the rodent lung migrating nematode *N. brasiliensis*. Infection with *N. brasiliensis* in mice elicits lung immune responses similar to that observed in allergic airway inflammation, thereby making it a valid model to study the interaction between IL-17A and Th2 inflammation.

**Results**: In this lung infection model, anti-Ym1 treatment led to a reduction in IL-17A expression and a reduction in the number of innate γδ T cells during the early stages of infection. The altered IL-17A response resulted in decreased IL-13 and IL-5 mRNA expression in whole lung tissue and reduced numbers of IL-5 + and IL-13 + CD4 + T cells and innate lymphoid cells; results mimicked by global IL-17A deficiency.

**Conclusion**: CLPs impaired the development of type 2 response by modulating IL-17 expression. This suggests that CLPs can regulate both innate and adaptive immune mechanisms that may be key in tipping the balance of airway inflammation towards neutrophil dominance during allergic lung pathology.

**Keywords**: Neutrophil, IL-17, Chitinase-Like Protein, Lung Inflammation

**References**:

1. Chupp GL et al. N Engl J Med. 2007;357:2016–27
2. Sutherland TE et al. Nat Immunol. 2014;15:1116–25

## POSTER DISCUSSION SESSION 2—Treatment

### P25 Once-daily tiotropium respimat add-on therapy improves lung function in patients aged 6–17 years with severe symptomatic asthma

#### E. Hamelmann^1^, C. Vogelberg^2^, S. Goldstein^3^, G. E. Azzi^4^, M. Engel^4^, R. Sigmund^5^, S. J. Szefler^6^

##### ^1^Children’s Center, Evangelisches Krankenhaus Bielefeld, and Allergy Center of the Ruhr University, Bochum, Germany; ^2^University Hospital Carl Gustav Carus, Technical University of Dresden, Dresden, Germany; ^3^Island Medical Research, Rockville Centre, New York, New York City, New York, United States; ^4^Boehringer Ingelheim Pharma GmbH & Co. KG, Ingelheim Am Rhein, Germany; ^5^Boehringer Ingelheim Pharma GmbH & Co. KG, Biberach An Der Riss, Germany; ^6^Children’s Hospital of Colorado and the University of Colorado School of Medicine, Aurora city, Colorado, United States

**Correspondence**: Eckard Hamelmann - eckard.hamelmann@evkb.de

*Clinical and Translational Allergy* 2017, **7(Suppl 2)**:P25

**Introduction**: Tiotropium Respimat (tioR) add-on therapy to inhaled corticosteroids (ICS) with or without additional controllers has been shown to improve lung function in Phase II and III studies of adults, adolescents and children with symptomatic asthma. We present a pooled analysis of lung function data in adolescents and children with severe symptomatic asthma.

**Method**: Two Phase III, randomised, double-blind, placebo-controlled, parallel-group, 12-week trials in patients aged 6–11 years (VivaTinA-asthma; NCT01634152) and 12–17 years (PensieTinA-asthma; NCT01277523) with severe symptomatic asthma. Patients received once-daily tioR 5 µg (2 × 2.5 µg), tioR 2.5 µg (2 × 1.25 µg) or placebo Respimat (pboR) as add-on to high-dose ICS plus another controller or as add-on to medium-dose ICS plus two other controllers. ICS dose was as defined in GINA 2009 (PensieTinA) or 2010 (VivaTinA) guidelines. Patients were required to have a ≥ 3-month (PensieTinA) or ≥ 6-month (VivaTinA) history of asthma and be symptomatic at screening and before randomisation by Asthma Control Questionnaire (interviewer-administered; VivaTinA) mean score ≥ 1.5. Primary end point of both studies: change from baseline (response) in peak forced expiratory volume in 1 s within 3 h post-dose (peak FEV_1(0–3h)_); key secondary end point: trough FEV_1_ response (measured 10 min before next dose of study medication); post hoc end point: trough FEV_1_/forced vital capacity (FVC) ratio, all measured at Week 12.

**Results**: 793 participants (VivaTinA: 401; PensieTinA: 392) were randomised across both trials; 792 were included in this pooled full analysis set. Baseline demographics and disease characteristics were balanced between treatment groups. TioR add-on therapy improved lung function in the pooled population at Week 12, with tioR 5 µg showing superior improvements in peak FEV_1(0–3h)_ response, trough FEV_1_ response and FEV_1_/FVC ratio versus pboR, and tioR 2.5 µg showing superior improvements in peak FEV_1(0–3h)_ response and FEV_1_/FVC ratio versus pboR, with numerical improvements in trough FEV_1_ response versus pboR (Table). Safety and tolerability of tioR in both trials was comparable to placebo.

**Conclusion**: Tiotropium Respimat add-on therapy is an effective bronchodilator, producing clinically meaningful improvements versus placebo in lung function in patients aged 6–17 years with severe symptomatic asthma, mirroring findings in adult patients with symptomatic asthma.

**Table:** Table 3 Treatment results

Response (change from baseline) measured at Week 12	Peak FEV_1(0–3h)_ response (mL)	Trough FEV_1_ response (mL)	Trough FEV_1_/FVC ratio (%)
Tiotropium Respimat 5 µg once daily (n = 260), adjusted mean of difference versus placebo Respimat ± SE (95% CI)	117 ± 34 (51, 183) p = 0.0005	71 ± 34 (3, 139) p = 0.0395	1.921 ± 0.666 (0.614, 3.229) p = 0.0040
Tiotropium Respimat 2.5 µg once daily (n = 263), adjusted mean of difference versus placebo Respimat ± SE (95% CI)	74 ± 33 (8, 140) p = 0.0273	64 ± 34 (−3, 132) p = 0.0617	1.930 ± 0.666 (0.623, 3.236) p = 0.0038

Full analysis set. CI, confidence interval; SE, standard error

**Keywords**: Asthma Management, Bronchodilators, Children

### P26 Near-fatal asthma: differential role of ECCO2R and VV-ECMO as rescue therapies

#### Raquel Mesquita^1^, Luis Coentrão^2^, Rui Veiga^2^, José-Artur Paiva^2^, Roberto Roncon-Albuquerque Jr^2^

##### ^1^Internal Medicine Department, Centro Hospitalar de São João, Porto, Portugal; ^2^Emergency and Intensive Care Medicine Department, Centro Hospitalar de São João, Porto, Portugal

**Correspondence**: Raquel Mesquita - raquelmsmesquita@gmail.com

*Clinical and Translational Allergy* 2017, **7(Suppl 2)**:P26

**Introduction**: During a near-fatal attack of asthma (NFA) alveolar hypoventilation underlies respiratory acidosis while severe hypoxemia indicates intrapulmonary shunt. Illustrate the differential role of low-flow extracorporeal CO_2_ removal (ECCO_2_R) and high-flow veno-venous extracorporeal membrane oxygenation (VV-ECMO) as rescue therapies in NFA.

**Method**: Patient 1 A 31-year-old male with early-onset asthma and epilepsy was admitted with acute respiratory failure (ARF) complicated by generalized tonic–clonic seizure. He presented severe acute respiratory acidosis without hypoxemia after intubation (pH-7.10 pCO_2_-82 mmHg P/F 556). Thoracic CT: diffuse bronchial wall thickness without atelectasis. On Day-5 dynamic hyperinflation (auto-PEEP 10 cmH_2_O) under deep sedation and neuromuscular blockade persisted, precluding weaning from invasive mechanical ventilation (IMV). ECCO_2_R was initiated. Extubation, active physical therapy and full patient mobilization were possible on Day-6. ECCO_2_R discontinuation, ICU and hospital discharges occurred on Day-8, -9 and -11, respectively.

**Results**: Patient 2 A 34-year-old obese female with late-onset and poorly controlled asthma was admitted with ARF with severe hypoxemia (pH-7.45 pCO_2_-30 mmHg P/F 69). After a trial of non-invasive ventilation, IMV was initiated. On Day-1, despite medical therapy and IMV optimizations, severe dynamic hyperinflation with refractory hypoxemia persisted and VV-ECMO was initiated. Thoracic CT revealed almost complete bilateral lung collapse. Methicillin-sensitive *S. aureus* and *S. pneumoniae* were isolated in endotracheal aspirate. Normal lung function was progressively reestablished following considerable viscid mucus secretions elimination and antibiotherapy. ECMO-VV was discontinued on Day-18. Extubation, ICU and hospital discharges occurred on Day-24, -27 and -55, respectively.

**Conclusion**: How this report contributes to current knowledge. During refractory NFA, low-flow ECCO_2_R allows correction of severe respiratory acidosis and facilitates extubation while high-flow VV-ECMO is needed when significant atelectasis complicates airway narrowing and severe hypoxemia ensues. Knowledge of respiratory failure pathophysiology in refractory NFA allows the correct use of different ECLS modalities as a bridge to recovery.

**Consent to publish**: Consent to publish was received from the patients.

**Figure:**
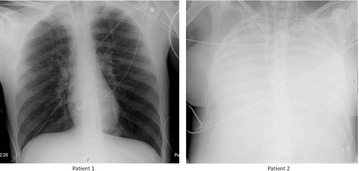
Figure 6 Patient 1 and patient 2

### P27 Clinical and functional characteristis of 4 severe asthmatic patients with absence of response to omalizumab

#### Wendy Vargas Porras, Ana González Moreno, Jesus Macías Iglesias, Gustavo Córdova Ramos, Yesenia Peña Acevedo, Miguel Angel Tejedor Alonso, Maria Del Mar Moro Moro

##### University Hospital Foundation Alcorcón, Madrid, Spain

**Correspondence**: Wendy Vargas Porras - wendy.drawenwen@gmail.com

*Clinical and Translational Allergy* 2017, **7(Suppl 2)**:P27

**Introduction**: 69.9% of patients with severe asthma respond to omalizumab, when it is used according to International Guidelines of asthma.

We describe clinical and functional characteristics of 4 patients with severe asthma treated with omalizumab and belong to our series of severe asthmatic patients treated with this drug.

**Method**: We collected 24 patients with severe asthma treated with omalizumab from the Allergy Unit of the University Hospital Foundation Alcorcón, during the period 2007 to 2015. Of these, 4 patients did not respond to treatment. The “No responders” was defined by the responsible doctor, based on the clinical and functional assessment of each patient considering several clinical and functional characteristics: beta-adrenergic use, number of exacerbations and scores ACQ and ACT, other comorbidities, spirometric and lung volumes. These values were measured in the last year before the start of omalizumab and the final year of treatment with omalizumab.

**Results**: In 4 patients it was withdrawn omalizumab because of no efficacy. Responders and non-responders were treated, at least, during 1 year. The non-responders had more non-respiratory comorbidities than responders (75%-25%,p = 0.07). However responders, seemed to have more severe asthma, with a greater number of exacerbations (responders 3.24 vs no responders 2.75), more use of beta-adrenergic (responders 2.47 vs no responders 1), more cycles oral corticosteroids (responders 2.64 vs no responders 1.5) and higher ACQ score (responders 2.1 vs no responders 1.8). Only use of oral corticosteroids and use of beta-adrenergic the differences were significant (p = 0.02 and 0.05 respectively). However the residual volume (RV) higher in non-responders (responders 188% vs no responders 154%), although differences were not statistically significant (p = 0.3).

In the last year of use of omalizumab, asthma was worse in the group of non-responders, with increased use of beta-adrenergic (p = 0.005) and greater use of steroids cycles (p = 0.002).

**Conclusion**: In non-responders patients, the RV was higher and also had more comorbidities.

The small number of our sample limits the validity of our findings.

### P28 One year follow-up of patients after omalizumab treatment withdrawal

#### Irena Krcmova, Jakub Novosad

##### Institute of Clinical Immunology and Allergy, University Hospital, Hradec Kralove, Czech Republic

**Correspondence**: Irena Krcmova - irena.krcmova@fnhk.cz

*Clinical and Translational Allergy* 2017, **7(Suppl 2)**:P28

**Introduction**: Omalizumab has demonstrated its clinical efficacy in asthmatic patients in number of studies. However, data about its clinical benefits after treatment cessation are still missing. To stress this challenging clinical issue, we have arranged a pilot observational-study following clinical and laboratory data of 12 patients with severe uncontrolled atopic asthma treated with omalizumab (11–61 months) for one year after treatment withdrawal due to clinical stability.

**Method**: Study had a mixed design with repeated measures at 4 time points, at treatment initiation, directly after treatment cessation, and 6 and 12 months thereafter. We have focused on clinical (pulmonary functions, inhalation corticosteroid (ICS) doses, asthma control test (ACT), skin prick tests positivity) and laboratory (FeNO, eosinophils and total IgE levels) parameters. Statistical analysis of collected data has been undertaken. Repeated measures were treated using a general linear model or Friedman ANOVA in case of normality assumption violation. Post-hoc analysis was applied using Wilcoxon signed-rank test with Bonferroni adjustment.

**Results**: We have demonstrated a significant reduction of ICS doses and SPT positivity (wheal diameter/mm) and increase of ACT during omalizumab treatment regardless of a dominant allergic linkage, treatment duration or omalizumab dose. This effect has been visible even 6 months after treatment withdrawal, but only ACT sustained increased significantly 12 months after treatment cessation. Two patients had a treatment of oral corticosteroids, which were permanently tapered during the omalizumab therapy and subsequently.

**Conclusion**: We conclude, that omalizumab treatment outcomes reach clinical and statistical significance even one year after treatment withdrawal. “Step down” of omalizumab in “treatment responders” remains a highly individual process that can undergo by mutual agreement with the patient under clearly defined treatment plan.

**Keywords**: Asthma, Omalizumab, Withdrawal, Clinical Effect

### P29 High FeNO leads to changes in asthma treatment: cohort analysis from the assessment of TH2 inflammation (ASTH2MA) survey

#### Nicola Alexander Hanania^1^, Marc Massanari^2^, Heike Hecker^2^, Eric Kassel^2^, Craig Laforce^3^, Kathy Rickard^4^

##### ^1^Baylor College of Medicine, Houston, Texas, United States; ^2^Circassia, Raleigh, North Carolina, United States; ^3^North Carolina Clinical Research, Raleigh, North Carolina, United States; ^4^Circassia, Raleigh, North Carolina, United States

**Correspondence**: Heike Hecker - heike.hecker@circassia.com

*Clinical and Translational Allergy* 2017, **7(Suppl 2)**:P29

**Introduction**: Recognizing TH_2_ inflammation in asthma has led to improvements in optimization of drug therapy and disease control. However, determination of inflammation using patient-based symptom assessment or spirometry is difficult and frequently underestimates asthma severity. Measurement of exhaled nitric oxide (FeNO) can support the clinical assessment of the patient at the point of care and can provide insights into underlying airway inflammation and therefore potentially help practitioners optimize their treatment decisions. High FeNO concentrations have been associated with more severe/uncontrolled asthma and poor compliance with ICS therapy.

**Method**: The objective of the ASTH_2_MA survey was to explore the real world impact of measuring FeNO on physicians’ treatment decisions. Physicians initially recorded their assessment of airway inflammation (low, intermediate or high) based on the patient’s clinical presentation and then by measuring patient’s FeNO using an approved electrochemical device. Based on the FeNO result, physicians recorded what changes in drug therapy were made.

**Results**: Data from 337 physician practices, which included 7,901 patients with asthma, were available for analysis. Assessment of inflammation using clinical impression vs FeNO measurement was determined to be low in 4,247 patients (53.8%) vs 5,083 (64.3%) (< 25 ppb); intermediate in 2,749 (34.8%) vs 1,802 (22.8%) (25–50 ppb) and high in 905 (11.5%) vs 1016 (12.9%) (> 50 ppb). Mean high FeNO was 87 ppb (range 51–300, median 77) with FeNO ≥ 51–99 ppb in n = 760, FeNO 100–199 ppb in n = 229 and FeNO 200–300 ppb in n = 27 patients. Clinical impression matched actual FeNO of < 25 ppb in 64.4%, 25–50 ppb in 46.9% and > 50 ppb in only 33.6%. Changes in treatment were made in 68.4% (695/1016) of patients who had a high FeNO (> 50 ppb); stepping up anti-inflammatory treatment in 96.1% and stepping down in 2.9%. Inhaled steroids were started in 345 patients and increased in 213 patients. Oral steroids were increased/started in 110 patients. Omalizumab was started in 2 patients.

**Conclusion**: Our results suggest that measurement of FeNO at point of care improves the clinical assessment of underlying airway inflammation in asthma and leads to clinically relevant changes in treatment especially in those patients presenting with high FeNO (> 50 ppb).

**Keywords**: FeNO, Asthma, Management, ASTH2MA Survey

### P30 4-month omalizumab efficacy outcomes for inadequate controlled severe allergic asthma in the Netherlands 2012–2015

#### Sanne Snelder, Gert-Jan Braunstahl

##### Sint Franciscus Gasthuis, Rotterdam, The Netherlands

**Correspondence**: Sanne Snelder - sannesnelder@hotmail.com

*Clinical and Translational Allergy* 2017, **7(Suppl 2)**:P30

**Introduction**: Since 2006, omalizumab has been prescribed for inadequately controlled severe allergic asthma in the Netherlands. In 2011, more stringent rules were applied by Dutch healthcare policy makers. This implied that omalizumab should be strictly prescribed within the registered label in exchange for reimbursement: inclusion criteria were a positive skin test or in vitro activity to a perennial aeroallergen, a FEV1 less than 80 percent, more than 2 severe exacerbations and substantial symptoms despite treatment with inhaled corticosteroids (ICS) and long-acting B2-agonists (LABAs).

To evaluate the 4-month efficacy outcomes of omalizumab treatment for inadequate controlled severe allergic asthma after the stringent rules were applied by the Dutch healthcare policy.

**Method**: This is a “real world” prospectively designed data registry in which the outcomes of patients (2012–2015) who received omalizumab were evaluated after the policy change. The primary endpoint was a good or excellent response to omalizumab after 16 weeks as scored by the treating physician. Secondary endpoints include change from baseline to week 16 in the Asthma Control Questionnaire (ACQ), FEV1 scores and oral corticosteroids (OCS) use.

**Results**: 403 patients had a complete data set and could be evaluated. 63% percent of the patients had a good or excellent response to omalizumab after 16 weeks. 85.5% of the responders showed more than 0.5 points improvement in the ACQ score at 16 weeks. The mean FEV increased from 71.58 to 79.06. 63% of the responders had an improvement of 5% of the FEV1. The maintenance OCS use was lower at 16 weeks. There was no relation between FEV1 < 80 and ≥ 80% at baseline and response after 4 months (Table 3). The response rate remained stable over the years 2012–2015 (Table 4). Figure 1 shows that most of the responders had an improvement of both FEV1 and ACQ. It also shows that ACQ appears to be a better measurement for a response than FEV1.

**Table:** Table 3 Response vs FEV1 < 80

	Response		
	YES	NO	Total
FEV1 < 80 YES	178	80	258
FEV1 < 80 NO	73	33	106
Total	251	113	364
	P = 0.981

**Table:** Table 4 Response vs year of inclusion

Year of inclusion	Response	No response
2012 (%)	63.6	36.4
2013 (%)	70.7	29.3
2014 (%)	64.9	35.1
2015 (%)	69.2	30.8
	P = 0.690	

**Conclusion**: 63% of the 403 patients with inadequately controlled severe allergic asthma had a good or excellent response to omalizumab after 16 weeks. Overall the ACQ improved, FEV1 increased and there was lower use of OCS at 16 weeks. There was no relationship between patients with a FEV1 < 80 and ≥ 80% at baseline and the response rate. Improvement of ACQ appears to be a better measurement for response than improvement of FEV1.

**Keywords**: Asthma, Omalizumab

**Figure:**
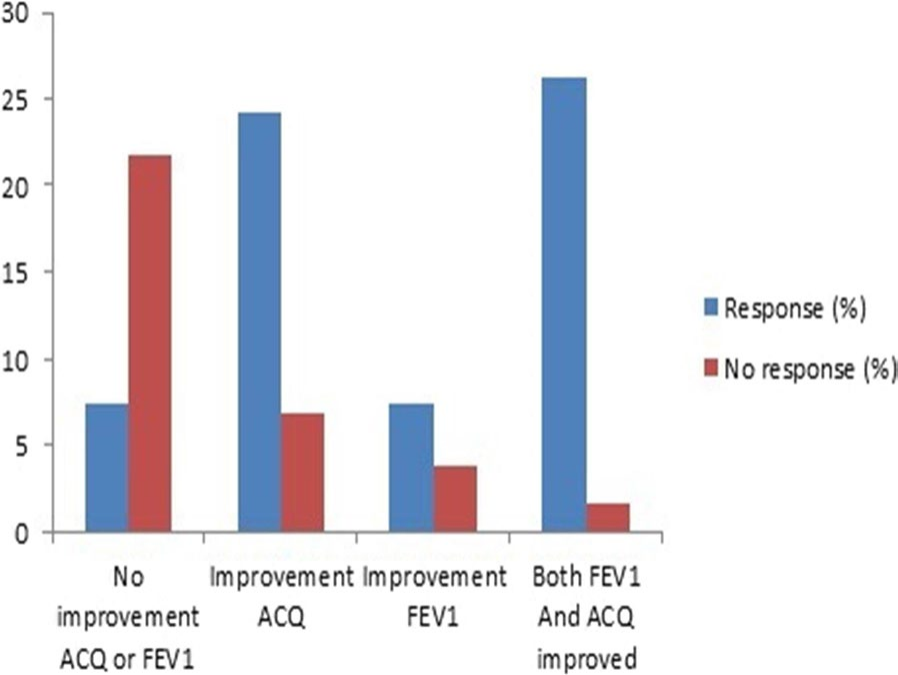
Figure 7 Improvement of ACQ and FEV1 compared to response

### P31 Adherence to oral corticosteroids; audit results of prednisolone assays

#### T. L. Jones, D. Neville, E. R. Heiden, E. Lanning, T. Brown, H. Rupani, K. S. Babu, A. J. Chauhan

##### Portsmouth Hospitals NHS Trust, Portsmouth, United Kingdom

**Correspondence**: Thomas Llewelyn Jones - thomas.jones@porthosp.nhs.uk

*Clinical and Translational Allergy* 2017, **7(Suppl 2)**:P31

**Introduction**: Oral corticosteroids (OCS) are used in the management of both acute and severe asthma. Acute courses are typically well tolerated, but well documented side effects occur in prolonged treatment. Side effects include skin thinning, cataracts, diabetes, osteopenia and easy bruising; these may impact adherence to treatment. Patients rarely admit poor adherence to treatment but, with increasing availability of monoclonal antibody therapy, adherence should be good prior to escalation to high-cost treatment. Mass-spectrometry can assess concentrations of cortisol and prednisolone in peripheral blood, and this can provide evidence of OCS adherence as well as assessing absorption. We conducted an audit of results of all our prednisolone assays conducted in patients with severe asthma.

**Method**: Data was collected on all patients with severe asthma undergoing prednisolone assays at our centre. OCS assays were performed prior to escalation of therapy where there was a suspicion of poor adherence. The results of the assay, participant age, gender, BMI, FEV1, Fractional Exhaled Nitric Oxide (FeNO), eosinophil count and prednisolone dose were recorded. These were used to determine which patient characteristics correlate with adherence.

**Results**: 14 adults taking oral prednisolone were included in this audit. This comprised 11 females and 3 males with a mean age of 46.3 ± 13.4 years, a mean prednisolone dose of 20.2 ± 10.4 mg, a mean BMI of 30.7 ± 7.2, a mean FeNO of 79.9 ± 53.2, mean FEV1 of 64 ± 17.6% predicted and a mean eosinophil count of 0.35 ± 0.34. 5 out of 14 were compliant as assessed by a supressed cortisol (5/5) or detectable blood prednisolone prior to dosing (4/5). All 14 patients absorbed prednisolone well following oral OCS administration. Factors correlating with compliance included gender (males 66% compliant, females 27% compliant), BMI (25.5 kg/m2 compliant vs 33.5, p = 0.05) and peripheral eosinophil count (compliant 0.15 vs 0.45, p = 0.09).

**Conclusion**: Only 36% of patients in our audit were adherent to OCS highlighting the importance of checking medication adherence during each clinical review, particularly when considering patients for novel therapies. Male gender, lower BMI and low eosinophil count were associated with compliance, but FeNO, FEV1 and OCS dose were not. We suggest an objective assessment of compliance prior to initiation of expensive novel biological agents.

**Keywords**: Prednisolone, Medication, Adherence, Education

### P32 Does the combination of omalizumab and Subcutaneous allergen immunotherapy (SCIT) have an add-on benefit to the efficacy and safety of allergen immunotherapy in asthma and allergic rhinitis? systematic review

#### M. Y. Eldegeir^1^, A. A. Chapman^2^, M. Ferwana^3^, M. Caldron^2^

##### ^1^National Guard Hospital, Dammam, Saudi Arabia; ^2^Imperial Collage London, London, United Kingdom; ^3^King Saud University, Riyadh, Saudi Arabia

**Correspondence**: Manal Yousif Eldegeir - almanjil@gmail.com

*Clinical and Translational Allergy* 2017, **7(Suppl 2)**:P32

**Introduction**: The last two decades witnessed a substantial expansion in our understanding of the pathoimmunological mechanism of allergic respiratory diseases. This led to a huge surge of interest in developing novel treatment modalities aiming for improved efficacy and safety of AIT. Co-administration of anti IgE is an attractive approach with the added benefit of omalizumab as a biological agent which has its own independent Immunomodulation effect on asthma and rhinitis.

The purpose of this review is to establish whether the combination of omalizumab and subcutaneous allergen immunotherapy (SCIT) has an add-on benefit to the efficacy and safety of SCIT in asthma and allergic rhinitis?

**Method**: This systematic review included double-blind randomized placebo controlled trials of the add-on benefits of the combination of omalizumab and SCIT compared to SCIT alone.

**Results**: The combination of omalizumab and SCIT has decreased symptoms score, rescue medication score, and symptoms load in patients with rhinitis and patients with rhinitis and co-existing asthma. In patients with asthma and those with rhinitis treated with rush immunotherapy, the percentage of patients developed systemic reaction to SCIIT including severe reaction requiring adrenaline administration was significantly decreased in the combination treatment group compared with SCIT/placebo group. Omalizumab has reduced the risk for anaphylaxis by 5 folds in rhinitis patients treated with rush immunotherapy protocol. Only one trial reported the of number of adrenaline doses used during the trial

**Conclusion**: The addition of omalizumab has increased the efficacy of SCIT in patients with rhinitis and patients with rhinitis and co-existing asthma. Omalizumab also reduced the risk of serious systemic reaction in patients with asthma and in rhinitis patients treated with rapid high dose of allergen. Addition of omalizumab has helped more patients at high risk of adverse effects related to SCIT to reach their target immunotherapy. An important findings of this review is that the use of adrenaline is under-reported by immunotherapy clinical trials

**Table:** Table 4 Systematic review

Trial	Kuehr et al 2002	Casale 2006	Kopp 2008	Massanari 2010
Disease	Seasonal rhinitis	Seasonal rhinitis	Seasonal rhinitis and asthma	persistent asthma
Severity	Moderate-severe	Moderate	mild asthma	At least moderate
Age range	6–17	18–50	11–46	18–55
Total number of patients	221	159	140	275
Site of study	Germany	USA	Germany	USA
Duration of enrolment	36 weeks	22 weeks	20 weeks	26 weeks
Allergen used	Grass and birch extract	aqueous short ragweed extract	Depigoid,modified grass pollen extract	Cat, dog and HDM extract
Protocol of immunotherapy	Conventional	Rush protocol	Pre-seasonal rush titration protocol	Cluster immunotherapy
Length of pre-seasonal immunotherapy	14 weeks	10 weeks	8 weeks	Perennial allergen
Time of administration of Omalizumab	12 week after immunotherapy started	Pre-treatment 9 weeks	Co-administration	Pre-treatment for 13 weeks
Dose of Omalizumab	0.016 mg/kg per IU/Ml	0.016 mg/kg/IgE IU/mL	Dose/kg/IgE IU/mL every 2–4 weeks	0.016 mg/kg/IgE IU/mL every 2 or 4 weeks

**Keywords**: Omalizumab, Immunotherapy, Asthma, Rhinitis, Allergic

